# Aberrant Cholesterol Metabolism and Wnt/*β*‐Catenin Signaling Coalesce via Frizzled5 in Supporting Cancer Growth

**DOI:** 10.1002/advs.202200750

**Published:** 2022-08-17

**Authors:** Shaoqin Zheng, Jiahui Lin, Zhongqiu Pang, Hui Zhang, Yinuo Wang, Lanjing Ma, Haijiao Zhang, Xi Zhang, Maorong Chen, Xinjun Zhang, Chao Zhao, Jun Qi, Liu Cao, Min Wang, Xi He, Ren Sheng

**Affiliations:** ^1^ College of Life and Health Science Northeastern University Shenyang 110819 P. R. China; ^2^ College of Sciences Northeastern University Shenyang 110004 P. R. China; ^3^ F.M Kirby Neurobiology Center Boston Children's Hospital Department of Neurology Harvard Medical School Boston MA 02115 USA; ^4^ Key Laboratory of Molecular Biophysics of the Ministry of Education National Engineering Research Center for Nanomedicine College of Life Science and Technology Huazhong University of Science and Technology Wuhan 430074 P. R. China; ^5^ School of Public Health Jilin University Changchun 130021 P. R. China; ^6^ Department of Cancer Biology Dana‐Farber Cancer Institute Department of Medicine Harvard Medical School Boston MA 02215 USA; ^7^ Institute of Translational Medicine Key Laboratory of Cell Biology of Ministry of Public Health and Key Laboratory of Medical Cell Biology of Ministry of Education Liaoning Province Collaborative Innovation Center of Aging Related Disease Diagnosis and Treatment and Prevention China Medical University Shenyang 110112 P. R. China; ^8^ Department of Biliary‐Pancreatic Surgery Affiliated Tongji Hospital Tongji Medical College Huazhong University of Science and Technology 1095 Jiefang Ave Wuhan 430030 P. R. China

**Keywords:** cholesterol, Frizzled receptor, pancreatic cancer, Wnt/*β*‐catenin signaling

## Abstract

Frizzled (Fzd) proteins are Wnt receptors and play essential roles in development, homeostasis, and oncogenesis. How Wnt/Fzd signaling is coupled to physiological regulation remains unknown. Cholesterol is reported as a signaling molecule regulating morphogen such as Hedgehog signaling. Despite the elusiveness of the in‐depth mechanism, it is well‐established that pancreatic cancer specially requires abnormal cholesterol metabolism levels for growth. In this study, it is unexpectedly found that among ten Fzds, Fzd5 has a unique capacity to bind cholesterol specifically through its conserved extracellular linker region. Cholesterol‐binding enables Fzd5 palmitoylation, which is indispensable for receptor maturation and trafficking to the plasma membrane. In Wnt‐addicted pancreatic ductal adenocarcinoma (PDAC), cholesterol stimulates tumor growth via Fzd5‐mediated Wnt/*β*‐catenin signaling. A natural oxysterol, 25‐hydroxylsterol competes with cholesterol and inhibits Fzd5 maturation and Wnt signaling, thereby alleviating PDAC growth. This cholesterol‐receptor interaction and ensuing receptor lipidation uncover a novel mechanism by which Fzd5 acts as a cholesterol sensor and pivotal connection coupling lipid metabolism to morphogen signaling. These findings further suggest that cholesterol‐targeting may provide new therapeutic opportunities for treating Wnt‐dependent cancers.

## Graphical abstract

The mechanism of Frizzled (Fzd) receptor regulation and aberrant cholesterol metabolism in pancreatic cancer are largely unknown. This study reveals that cholesterol specifically binds to Fzd5 and enables Fzd5 maturation to activate Wnt/*β*‐catenin signaling which is required for pancreatic cancer. Therefore, Fzd5 crucially coalesces cholesterol metabolism and Wnt/*β*‐catenin signaling, and oxysterol provides new therapeutic promise in cancer treatment.

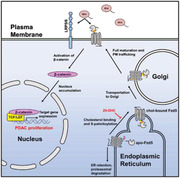

## 1 Introduction

Wnt/*β*‐catenin signaling is a well‐conserved signaling cascade in metazoan and plays a pivotal role in embryonic development and adult tissue homeostasis.^[^ ^1^ ^]^ As Wnt receptors, ten Frizzled (Fzd) subtypes exist in mammals.^[^ ^2^ ^]^ These subtypes are relatively conserved for their cysteine‐rich domain (CRD) and 7‐transmembrane (7‐TM) region, but less conserved for the linker region in between. Structural studies suggest that the linker region is flexible and may vary among different Fzds.^[^ ^3^ ^]^ Despite potential functional redundancy, Fzd subtypes were reported to have irreplaceable functions in different biological processes. Thus, Fzd4 is required for retinal vascular development and can uniquely bind to the Norrin ligand.^[^ ^4^ ^]^ Fzd7 is critically involved in intestinal stem cell maintenance while Fzd5 has a key role in driving the growth of a subtype of pancreatic ductal adenocarcinoma (PDAC) that harbors mutations of the tumor suppressor gene RNF43, which encodes an E3 ligase for Fzd degradation.^[^ ^5^ ^]^ In terms of regulation, a myriad of publications reported that the ubiquitination apparatus including RNF43 and related ZNRF3, USP8, TMEM79, and others critically modulate Fzd stability.^[^ ^6^ ^]^ Additionally, fatty acids have been found residing in the Fzd1/2/7 CRD domain and play roles in Fzd maturation and possibly dimerization, suggesting a tantalizing link by which biogenesis of the Fzd1/2/7 subfamily may be subjected to regulation by fatty acid metabolism.^[^ ^7^ ^]^ Though progress has been made in the past decade, further understanding of function, biogenesis, and regulatory mechanism of individual Fzd remains critical but challenging.

Cholesterol composes cell membranes as basic building blocks and serves as an energy source in cells.^[^ ^8^ ^]^ It can be transported into cells via endocytosis of lipoproteins or synthesized de novo from Acyl‐CoA in the ER. Besides these classical functions, cholesterol has also been shown in recent years to act as a signaling molecule that directly participates in signaling cascades.^[^ ^9^ ^]^ For example, through direct binding to Smoothened (Smo), which is a distant serpentine protein in the Fzd family, cholesterol promotes the localization of Smo into primary cilia and allosterically activates Hedgehog (Hh) signaling even in the absence of a Hh ligand.^[^ ^10^ ^]^ However, based on previous biochemical and structural studies of CRDs, it was argued that no Fzd subtype is capable of cholesterol binding due to the steric hindrance.^[^ ^10a,b^ ^]^ As natural or synthetic products, oxysterols may reduce the cellular cholesterol level or block its binding to partner proteins through competition, and therefore possess promises in treating cholesterol‐related human diseases.^[^ ^9^ ^]^

As one of the deadliest cancers, PDAC patients have a dismal 5‐year survival rate of less than 10%.^[^ ^11^ ^]^ Though initially driven by oncogenic KRAS mutations, aberrant Wnt/*β*‐catenin signaling activation contributed substantially to PDAC progression.^[^ ^12^ ^]^ Loss‐of‐function mutations of RNF43 have been frequently observed in PDAC, and growth of RNF43‐mutant PDAC was reported to be Wnt‐ and Fzd5‐dependent.^[^ ^5^, ^13^ ^]^ Interestingly, PDAC demands high levels of cholesterol metabolism and is particularly sensitive to perturbation of cellular cholesterol levels.^[^ ^14^ ^]^ The molecular basis of this unusual cholesterol‐dependence exhibited by PDAC is poorly understood. Epidemiological studies have found that cholesterol‐lowering drugs such as statins promote higher survival rates of PDAC patients, though it remains uncertain whether statins function by lowering the cholesterol level.^[^ ^15^ ^]^

Here we report a surprising finding that cholesterol specifically binds to a unique Fzd subtype Fzd5. This binding mechanism is distinct from fatty acid binding to Fzd1/2/7 or sterol binding to Smo in that cholesterol interacts with a unique and conserved extracellular linker/loop region of Fzd5 other than its CRD, enabling subsequent S‐palmitoylation of Fzd5 on a conserved cysteine residue of the carboxyl‐terminal tail. This palmitoylation is seen in Fzd subclass receptors for the first time and is indispensable for Fzd5 protein trafficking and maturation to the plasma membrane (PM). Functionally, the cellular level of cholesterol pivotally regulates Fzd5‐mediated Wnt/*β*‐catenin signaling activity. A point mutation of either the cholesterol‐binding site or the palmitoylation site abolished Fzd5 trafficking to the PM and hence Wnt signal transduction. In RNF43‐mutant PDAC, the cholesterol level governs tumor growth through Fzd5‐mediated Wnt/*β*‐catenin signaling in vitro and in vivo. 25‐hydroxyloxysterol (25‐OHC) competes with cholesterol binding to Fzd5 causing PDAC growth stagnancy by inhibiting Wnt/*β*‐catenin signaling. Taken together, we have uncovered a novel mechanism of cholesterol binding and palmitoylation of Fzd5 that controls the growth of RNF43‐mutant PDAC, and revealed that oxysterols may possess PDAC therapeutic potential by blocking Fzd5‐cholesterol interaction. These findings suggest that Fzd5 acts as a cholesterol sensor and master regulator to couple lipid metabolism to Wnt signaling.

## 2 Results

### 2.1 Cholesterol Specifically and Reversibly Binds to Fzd5, Depending Primarily on Conserved Residues at the Extracellular Linker and Loop

**Figure 1:**
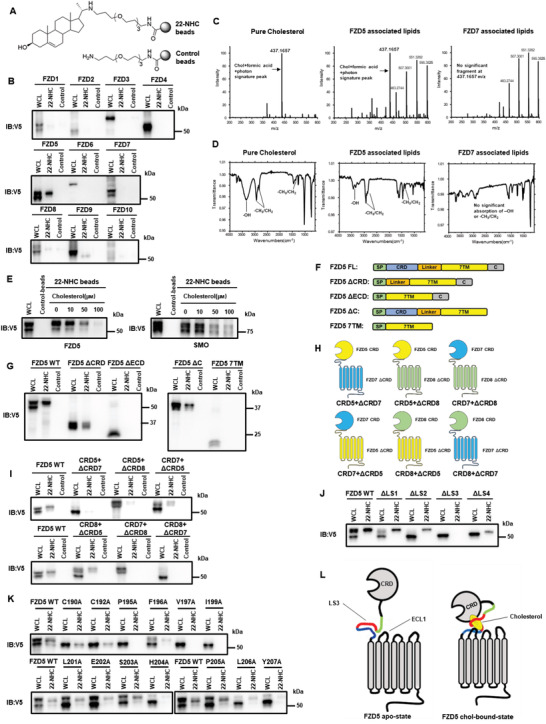
Cholesterol specifically and reversibly binds to Fzd5, depending primarily on conserved residues at the extracellular linker and loop. A) Demonstration of synthesized 22‐NHC beads and control beads. B) In vitro pulldown assay and WB of Fzd1‐10 by 22‐NHC or control beads. C) Mass‐spectrometry detection of Fzd5‐bound cholesterol (m/z range: 200–600). The cholesterol signature peak at m/z 437.1657 (combined particle of cholesterol, formic acid, and photon) was clearly seen in Fzd5‐associated lipids. Pure cholesterol and Fzd7‐associated lipids are used as positive and negative controls, respectively. D) FT‐IR showing the stretching vibration of ‐OH and ‐CH_3_/CH_2_ and the bending vibration of ‐CH_3_/CH_2_ (cholesterol signature IR absorption) were clearly seen in Fzd5‐associated lipids. Pure cholesterol and Fzd7‐associated lipids are used as positive and negative controls, respectively. E) 22‐NHC beads pulldown and competition assay of Fzd5 and Smo by cholesterol as a competitor. F) Schematic of various Fzd5 truncation constructs. FL: full‐length; ΔCRD: deletion of CRD; ΔECD: deletion of extracellular domain; ΔC: deletion of the carboxyl terminus; 7TM: transmembrane domain only. G) 22‐NHC beads pulldown assay of Fzd5 truncation constructs depicted in (F). H) Schematic of CRD‐swapped chimeric Fzds. (i.e.: CRD5+ΔCRD7 represents the chimeric protein consisting of Fzd5 CRD and Fzd7 ΔCRD.) I) 22‐NHC beads pulldown assay of the chimeric Fzds depicted in (H). J) 22‐NHC beads pulldown assay of Fzd5 full‐length or truncation of linker segment 1–4 (ΔLS1‐4). K) 22‐NHC beads pulldown assay of Fzd5 WT and point‐mutations of all conserved residues on LS3. L) Working model of cholesterol binding to Fzd5: owing to the steric hindrance, cholesterol could not insert into the hydrophobic grove in the CRD as PA does; the exposed hydrophobic part of cholesterol appears to be covered up by the hydrophobic and aromatic residues in the extracellular linker and loop regions, which form a “cove” with CRD possibly acting as “cap” to wrap around cholesterol. WCL: whole cell lysate.

The Fzd family is a unique protein branch of the Class F GPCR that contains an extracellular CRD. This class includes ten Fzd subtypes and Smo. The Smo CRD has been shown to bind to sterols in biochemical assays and structural studies.^[^ ^10a,b,d^ ^]^ By comparing Fzd and Smo CRD structures, it is suggested that the hydrophobic groove of Fzd CRD only allows docking of fatty acids but not bulky sterols (Figure S1A, Supporting Information).^[^ ^10b^ ^]^ Since there are ten different Fzd subtypes, we decided to experimentally test if any Fzd subtype might be capable of sterol binding. To achieve reliable results, we followed the exact strategy developed by Nedelcu et al. and synthesized the orthogonal 22‐NHC (22‐azacholesterol) beads as an appreciated chemical biological tool for studying cholesterol binding in vitro (**Figure** **1**A).^[^ ^10a^ ^]^ Binding results show that indeed most Fzd subtypes do not harbor binding capacity to 22‐NHC beads (Figure 1B). Surprisingly, however, Fzd5 uniquely displays strong binding to the 22‐NHC beads (Figure 1B). This binding was further consolidated by analytical chemical approaches. Both Mass‐spectrometry and Fourier‐transformed Infrared (FT‐IR) confirmed that Fzd5 binds to cholesterol in vivo (Figure 1C, D; Figure S1B, Supporting Information). We then tested whether cholesterol binding to Fzd5 was specific and reversible. Using Smo as a control, we performed a competition assay by adding free cholesterol as a competitor in the assay.^[^ ^10a^ ^]^ Either Fzd5 or Smo was competed off 22‐NHC beads by cholesterol dose‐dependently (Figure 1E).

Based on the findings above, we sought to define the cholesterol‐binding site of Fzd5. We constructed several Fzd5 deletion mutants for the purpose, including deletion of CRD (ΔCRD), the entire extracellular domain (ΔECD), deletion of the carboxyl terminus (ΔC) and a mutant with transmembrane domains only (7TM) (Figure 1F). In 22‐NHC pulldown assays, ΔECD and 7TM lacked any cholesterol binding capacity, whereas ΔCRD and ΔC retained significant cholesterol binding capacity (Figure 1G). These data suggest that Fzd5 ECD but not CRD is essential for cholesterol‐binding. This result contrasts that of Smo CRD, which is necessary and sufficient for cholesterol‐binding,^[^ ^10a,b,f^ ^]^ suggesting distinct mechanisms of cholesterol‐binding by Smo and Fzd5. Also, an apparent difference can be seen between cholesterol and fatty acid binding to Fzd, since the latter requires CRD alone.

We also constructed a series of chimeric proteins by swapping the CRD among Fzd5, Fzd7, and Fzd8 (Figure 1H). For instance, we named the chimeric protein consisting of Fzd5 CRD and Fzd7 ΔCRD as “CRD5+ΔCRD7” (the rest followed the same rule). Fzd5 and Fzd8 belong to the same Fzd subfamily and have almost identical CRDs, but they differ significantly in the extracellular linker region connecting CRD and 7TM. Using these chimeric Fzds, we found that fusing CRD of either Fzd7 or Fzd8 onto Fzd5 ΔCRD displayed strong binding to cholesterol, whereas the chimeric protein with Fzd5 CRD plus Fzd7 or Fzd8 ΔCRD showed very weak binding to 22‐NHC beads (Figure 1I). On the other hand, when we used the whole ECD of Fzd5, Fzd7, and Smo in the 22‐NHC pulldown assay, Fzd5 and Smo ECD but not Fzd7 ECD showed strong cholesterol‐binding that appears to be similar to the full‐length Fzd5 and Smo (Figure S1C, Supporting Information). Together, these results suggest that CRD of Fzd5 is not sufficient for binding to cholesterol and the extracellular linker region may play a major role in this process.

Next, we compared the sequences of the linkers of all Fzd subtypes. The alignment showed that the linker region of Fzd5 was distinct from other subtypes (Figure S1D, Supporting Information). We then aligned the sequences of Fzd5 linker region among vertebrates and arbitrarily separated it into four segments, named as linker segment 1–4 (LS1‐4) (Figure S1E, Supporting Information). LS3/4 but not LS1/2 is conserved among vertebrate Fzd5 proteins. We generated Fzd5 deletion mutants that delete each segment and were named as ΔLS1‐4 (deletion of linker segment 1–4) and the binding of these truncations to 22‐NHC beads was examined. It was shown that only ΔLS3 completely lost the binding capacity while other mutants retained strong binding (Figure 1J). We further broke down LS3 into four smaller segments and constructed the truncation of each, named ΔLS3‐1 to ΔLS3‐4 (Figure S1F, Supporting Information). ΔLS3‐2, ΔLS3‐3, and ΔLS3‐4 lost the cholesterol binding, which was further confirmed by Ala substitution mutants (Figure S1G–I, Supporting Information). There are several conserved residues containing hydrophobic or aromatic side chains in this region, and we reasoned that these residues could be directly involved in binding cholesterol given their hydrophobic or aromatic nature. We thus mutated these residues to Ala individually. As expected, several of these mutants showed a loss‐of‐function effect on cholesterol binding (Figure 1K). In particular, a single alteration such as I199A resulted in complete loss. Based on the structural model, we also found a few conserved hydrophobic/aromatic residues on the extracellular loop1 (ECL1) that are in close proximity with the ones in LS3 and we showed that point‐mutation of H304/I305 to Ala leads to loss of cholesterol binding (Figure S1J–L, Supporting Information). In sum, our results suggest that conserved hydrophobic and aromatic residues at LS3 and ECL1 are mainly responsible for Fzd5‐cholesterol binding (Figure 1L). Mutations of these critical residues can individually lead to the abolishment of Fzd5‐cholesterol binding. These results agree with the previous claim that Fzd CRD cannot accommodate cholesterol due to the steric hindrance, but argue against the notion that Fzd receptor is incapable of cholesterol binding.

### 2.2 Cholesterol Regulates Fzd5 Protein Levels at the PM and Fzd5‐Mediated Wnt/*β*‐Catenin Signaling

**Figure 2:**
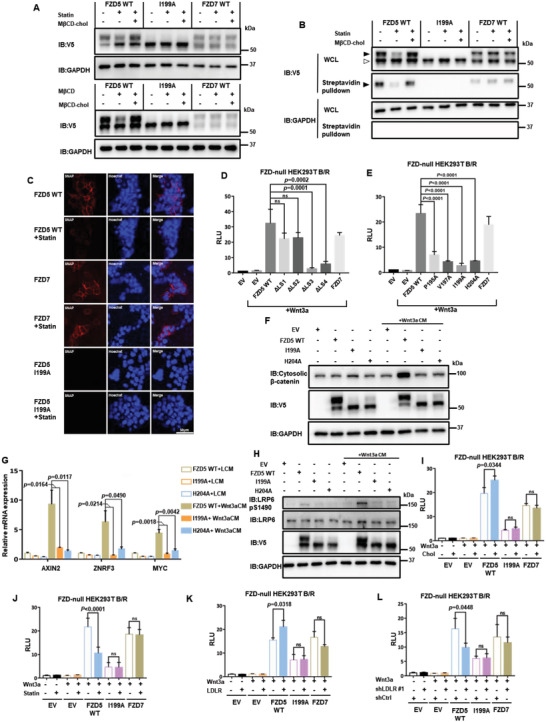
Cholesterol regulates Fzd5 protein levels at the PM and Fzd5‐mediated Wnt/*β*‐catenin signaling. A) Upper panel shows the mature (upper) band and the immature (lower) band distribution of V5‐tagged Fzd5 WT, I199A, and Fzd7 under normal culture, cholesterol starvation by statin, and rescue conditions in HEK293T cells. Lower panel shows acute cholesterol depletion by M*β*CD and rescue in HEK293T cells. B) Surface biotin labeling assay of Fzd5 WT, I199A, and Fzd7 under normal, cholesterol starvation (by statin treatment) and rescue conditions (by M*β*CD‐cholesterol complex treatment). Solid arrow head: mature Fzd; hollowed arrow head: immature Fzd. C) Confocal fluorescent imaging of SNAP‐tagged Fzd5, Fzd7, and Fzd5 I199A on the plasma membrane under normal and statin treatment conditions in HEK293T cells. SNAP‐tagged proteins are labelled with cell‐impermeable SNAP‐Surface549. Hoechst labels the nucleus. All images in the panel are in the same scale. D) TOPFlash assay in Fzd‐null HEK293T while transfecting Fzd5 WT, ΔLS1‐4 mutants, and Fzd7. Error bars mean ± SD, n = 3 replicates, by one‐way ANOVA analysis. E) TOPFlash assay in Fzd‐null HEK293T while transfecting Fzd5 WT, Fzd5 cholesterol binding loss‐of‐function point mutations, and Fzd7. Error bars mean ± SD, n = 3 replicates, by one‐way ANOVA analysis. F) Cytosolic *β*‐catenin assay of Fzd5 WT, I199A, and H204A under LCM or Wnt3a CM treatment in Fzd‐null HEK293T cells. G) RT‐qPCR assay of Wnt target gene AXIN2, ZNRF3, and MYC in Fzd‐null HEK293T cells. Error bars mean ± SD, n = 3 replicates, by one‐way ANOVA analysis. H) LRP6 phosphorylation assay of Fzd5 WT, I199A, and H204A under LCM or Wnt3a CM treatment in Fzd‐null HEK293T cells. I,J) TOPFlash assay in Fzd‐null HEK293T while transfecting Fzd5 WT, I199A, and Fzd7 under cholesterol addition (I) or cholesterol starvation treatment (J). Error bars mean ± SD, n = 3 replicates, by two‐tailed unpaired student's t‐test analysis. K,L) TOPFlash assay in Fzd‐null HEK293T while transfecting Fzd5 WT, I199A, and Fzd7 under LDLR overexpression (K) or knockdown (L) conditions. Error bars mean ± SD, n = 3 replicates, by two‐tailed unpaired student's t‐test analysis. RLU: relative luciferase unit. ns: not significant.

Next, we sought to interrogate the cellular and functional significance of Fzd5‐cholesterol interaction. Based on previous studies of ectopic Fzd5 expression, newly synthesized/immature Fzd5 in the ER and matured Fzd5 at the PM can be distinguished molecularly by gel electrophoresis, in which two discrete Fzd5 protein bands are observed.^[^ ^6^, ^16^ ^]^ The mature band (slower‐migrating) represents the glycosylated protein at the PM for ligand engagement; whereas the immature and (faster‐migrating) form represents newly synthesized protein in the ER yet to be fully glycosylated. Surface biotinylation only labeled the upper mature Fzd5 band at the PM but not the lower immature band in the ER (Figure S2A, Supporting Information). When we inspected all the Fzd5 mutants defective in cholesterol‐binding including V197A, I199A, H304A/I305A, and etc., we realized all of them lost the upper band in common (Figure 1I; Figure S1K, Supporting Information). Therefore, we hypothesized that cholesterol binding could affect the mature Fzd5 level at the PM. Thus, we perturbed the cellular cholesterol level by statin (long‐term cholesterol starvation), methyl‐*β*‐cyclodextrin (M*β*CD) treatment (acute cholesterol deprivation), or M*β*CD‐cholesterol complex (cholesterol supplementation). As expected, decreased cellular cholesterol levels either by statin or M*β*CD resulted in a significant reduction of the upper mature band of Fzd5. While in cells fed with additional cholesterol, the mature band re‐appeared and became more prominent (**Figure** **2**A). For comparison, Fzd7 was affected by none of the cholesterol perturbations (Figure 2A). These data suggest that the cellular cholesterol level is required for and elevates the Fzd5 level at the PM but not that of Fzd7. This conclusion was further supported by subcellular fractionation experiments (Figure 2B; Figure S2B, Supporting Information). Fzd5 mutants defective in cholesterol‐binding such as I199A exhibited primarily or only the lower band regardless of cholesterol depletion or repletion and were not labeled by surface biotinylation (Figure 2A, B; Figure S2C, Supporting Information). We also employed SNAP‐tag labeling to detect intracellular and PM‐localized Fzd5 based on a cell‐permeable or ‐impermeable dye, respectively. This assay showed that the cholesterol level was positively correlated with the surface level of Fzd5 but not Fzd7, whereas Fzd5 I199A was hardly detected on the cell surface, consistent with results by biochemical analyses (Figure 2C; Figure S2D, Supporting Information). These results together show that cholesterol binding critically regulates Fzd5 level at the PM.

We next examined the role of cholesterol in Fzd5‐mediated Wnt/*β*‐catenin signaling. Given the functional redundancy among Fzds, we used Fzd1‐10 knockout HEK293T (Fzd‐null) cell (gifted by Dr. Feng Cong)^[^ ^17^ ^]^ and expressed Fzd5 or its mutants to study their capacity as a Wnt receptor by functional complementation. As we reported,^[^ ^17^ ^]^ the Fzd‐null cell did not respond to Wnt3a protein in the TCF‐responsive luciferase reporter (TOPFlash) assay. The Wnt3a response was restored by expression of Fzd7, Fzd5, or Fzd5 ΔLS1, ΔLS2 but not the ΔLS3 or ΔLS4 mutant (Figure 2D; Figure S2E, Supporting Information). Importantly, Fzd5 single residue mutants (in LS3) that are defective in cholesterol‐binding also failed to restore the Wnt3a response, consistent with their absence at the PM (Figure 2E; Figure S2F, Supporting Information). These results were further confirmed by cytosolic *β*‐catenin accumulation, Wnt target gene expression, and LRP6 phosphorylation upon Wnt3a treatment (Figure 2F–H). When we chemically perturbed the cholesterol level by statin or M*β*CD‐cholesterol complex or genetically modulated cholesterol level by knocking down or overexpression of LDLR (low‐density lipoprotein receptor), we found that Fzd5‐mediated Wnt/*β*‐catenin signaling was positively correlated with cellular cholesterol level (Figure 2I–L; Figure S2G–I, Supporting Information). Herein, we conclude that cholesterol specifically modulates Fzd5‐mediated Wnt/*β*‐catenin signaling via the regulation of Fzd5 protein level in the PM.

### 2.3 Cholesterol Regulates Fzd5 Maturation and Stability Independent of the Ubiquitination‐Lysosomal Degradation Pathway

**Figure 3:**
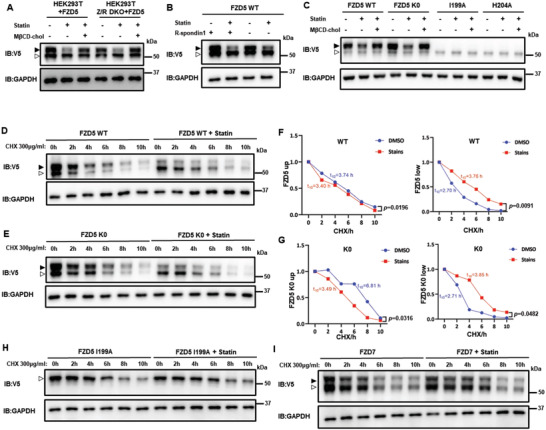
Cholesterol regulates Fzd5 maturation and stability independent of ubiquitination‐lysosomal degradation pathway. A) The mature/immature band distribution of Fzd5 in HEK293T or HEK293T Z/R DKO under normal, statin treatment and cholesterol rescue conditions. B) The mature/immature band distribution of Fzd5 under the treatment of statin or R‐spondin1 in HEK293T cells. C) The mature/immature band distributions of Fzd5 WT, K0, I199A, and H204A under normal, statin treatment and cholesterol rescue conditions in HEK293T cells. D,E) Pulse‐chase assay of Fzd5 WT (D) and K0 (E) under DMSO or statin treatment in HEK293T cells. F,G) The quantification by ImageJ of the upper/lower protein and the half‐lives in (D) and (E), respectively. By two‐tailed unpaired student's t‐test analysis. H,I) Pulse‐chase assay of Fzd5 I199A (H) and Fzd7 (I) under DMSO or statin treatment. Solid arrow head: mature Fzd; hollowed arrow head: immature Fzd. ns: not significant.

Fzd5 protein expression at the PM, where it functions as a Wnt receptor, involves biogenesis and protein stability regulation via the ubiquitination‐lysosomal degradation pathway.^[^ ^5^, ^6^ ^]^ We investigated whether cholesterol binding regulates Fzd5 maturation to the PM or the clearance from the PM. ZNRF3 and RNF43 (Z/R) are E3 ubiquitin ligases that ubiquitinate Fzd at the PM. In the presence of Z/R, Fzd is ubiquitinated and endocytosed, thereby undergoing lysosomal degradation.^[^ ^6a,b^ ^]^ To test whether cholesterol affects surface Fzd5 level via Z/R, we used Z/R double knockout (Z/R DKO) HEK293T cell.^[^ ^18^ ^]^ When we perturbed cholesterol levels as described above in the DKO cells, the mature Fzd5 still displayed significant reduction upon cholesterol starvation and recovered by exogenous cholesterol supplementation (**Figure** **3**A). Treatment of Rspo1, an antagonist of Z/R,^[^ ^6a,b^ ^]^ had no effect on statin‐induced reduction of the Fzd5 PM level (Figure 3B). These data together suggest that cholesterol regulation of Fzd5 at the PM is independent of Z/R. To examine if cholesterol could affect Fzd5 level through ubiquitination by other E3 ligases, we substituted all Lys into Arg in the intracellular regions of Fzd5 (K0) based on the previous report.^[^ ^6d^ ^]^ K0 mutant indeed exhibited higher protein levels and insensitivity to lysosomal inhibitor treatment (Figure S3A, Supporting Information). But the mature form of K0 mutant still diminished during cholesterol starvation, which was recovered by cholesterol addition (Figure 3C); this effect was not due to the alteration of Fzd5 endocytosis by cholesterol perturbation (Figure S3B, Supporting Information). Further, we evaluated the protein stability and maturation by pulse‐chase experiments in which we blocked the de novo protein synthesis by cycloheximide (CHX) and tracked the time‐lapse behavior of the existing proteins (Figure 3D, E). We were able to visualize the disappearance of the immature Fzd5 band and a concurrent increase of the mature Fzd5 band, reflecting Fzd5 maturation to the PM (Figure 3D, E). During cholesterol starvation, the immature bands of both the WT and the K0 mutant persisted for a much longer period, likely resulting from an inability to maturate to the PM (Figure 3D–G). In the same pulse‐chase experiment, maturation of cholesterol‐binding defective Fzd5 I199A did not occur whereas Fzd7 maturation proceeded regardless of cholesterol depletion (Figure 3H, I; Figure S3C, D, Supporting Information). On the other hand, we monitored the maturation of Fzd5 under cholesterol supplementation. As expected, the maturation of the WT Fzd5 became faster as shown by the band migration while I199A remained unaltered (Figure S3E, F, Supporting Information). Taken together, these results suggested that cellular cholesterol critically regulated Fzd5 maturation. And this regulation is independent of ubiquitination‐lysosomal degradation pathway.

### 2.4 Cholesterol Binding Enables Fzd5 S‐Palmitoylation, Which is Required for Fzd5 Maturation

**Figure 4:**
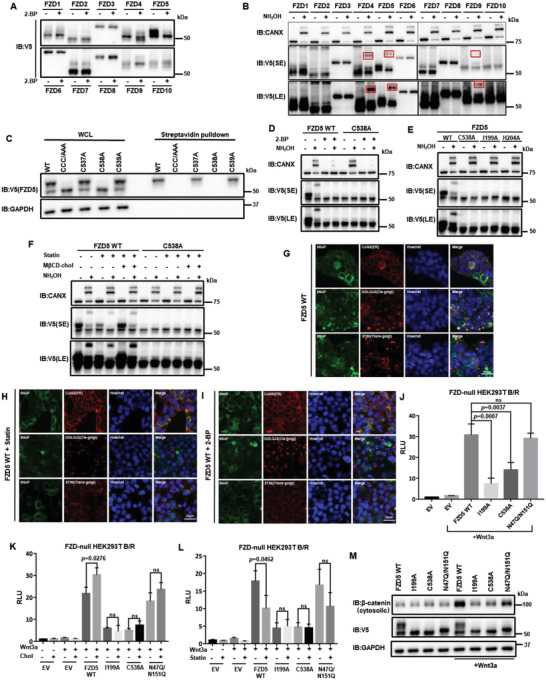
Cholesterol binding enables Fzd5 S‐palmitoylation, which is required for Fzd5 maturation. A) The band patterns of Fzd1‐10 under 2‐bromopalmitate (2‐BP) treatment in HEK293T cells. B) APE‐assay of Fzd1‐10 for palmitoylation detection in HEK293T cells. Among 10 subtypes, Fzd4, Fzd5, and Fzd9 showed clear band migration due to palmitoylation (highlighted by red boxes). C) Surface biotin labeling of the Cys mutations at Fzd5 cytoplasmic tail. CCC/AAA: C537A/C538A/C539A. D) APE‐assay of Fzd5 WT and C538A under the treatment of 2‐BP in HEK293T cells. E) APE‐assay of Fzd5 WT, C538A, I199A, and H204A in HEK293T cells. F) APE‐assay of Fzd5 WT and C538A under normal, statin treatment and cholesterol rescue conditions in HEK293T cells. G–I). Fluorescent microscopic images showing Fzd5 WT subcellular localizations under normal (G), statin treatment (H), and 2‐BP treatment (I) conditions in HEK293T cells. SNAP‐Fzd5 is labelled by SNAP Cell Oregon Green. CANX (ER), GOLGA2 (*cis*‐Golgi) and STX6 (*trans*‐Golgi) are immunofluorescent. All images are in the same scale. J) TOPFlash assay in Fzd‐null HEK293T while transfecting Fzd5 WT, I199A, C538A, and N47Q/N151Q. Error bars mean ± SD, n = 3 replicates, by one‐way ANOVA analysis. K,L) TOPFlash assay in Fzd‐null HEK293T while transfecting Fzd5 WT, I199A, C538A, and N47Q/N151Q under additional cholesterol treatment (K) or statin treatment (L). Error bars mean ± SD, n = 3 replicates, by two‐tailed unpaired student's t‐test analysis. M) Cytosolic *β*‐catenin assay of Fzd5 WT, I199A, C538A, and N47Q/N151Q under LCM or Wnt3a CM treatment in Fzd‐null HEK293T cells. SE: short exposure. LE: long exposure. RLU: relative luciferase unit. ns: not significant.

From the abovementioned results, we exclude the possibility that cholesterol regulates Fzd5 through ubiquitination‐lysosomal degradation. Our results suggest that cholesterol regulates Fzd5 maturation and trafficking. It was well‐documented that N‐glycosylation critically regulates the maturation and trafficking of transmembrane proteins. Based on the previous report and prediction (Figure S4A, Supporting Information), two N‐Glycosylation sites (N47 and N151) are present in Fzd5, yet mutations on these sites do not affect Fzd5 transportation and function.^[^ ^16^ ^]^ On the other hand, protein S‐palmitoylation was known to regulate receptor trafficking such as ER exit.^[^ ^19^ ^]^ To test whether Fzd5 is palmitoylated, we treated the cells with 2‐bromopalmitate (2‐BP) to block protein palmitoylation. 2‐BP treatment resulted in a significant reduction of the mature Fzd5 band, suggesting the possibility of Fzd5 palmitoylation (**Figure** **4**A). Further, we employed the APE (Acyl‐PEG Exchange) assay to accurately detect protein palmitoylation based on the molecular weight shift of the palmitate surrogate.^[^ ^20^ ^]^ In brief, the free cysteine was capped with NEM (N‐Ethylmaleimide), as the palmitoylated cysteine was reduced by NH_2_OH and then conjugated by mPEG‐Mal (Methoxypolyethylene glycol maleimide) to achieve specific migration on gel electrophoresis. The mature Fzd5 band showed a clear change in mobility, indicating Fzd5 palmitoylation (Figure 4B), which was substantiated via click chemistry by employing alkyne‐tagged palmitic acid analog (Figure S4B, Supporting Information). We therefore searched for all conserved cysteines on Fzd5 to identify the S‐palmitoylation site. Most of the cysteines are located extracellularly and form disulfide bonds based on the previous reports.^[^ ^3^ ^]^ However, protein S‐palmitoylation usually occurs on the cytoplasmic side. We thus focused on intracellular cysteines (three in‐a‐row) of Fzd5, which are unique among all Fzd subtypes and conserved in mammals (Figure S4C, D, Supporting Information). It was shown that mutation of one of the three cysteines, C538A, resulted in the loss of a mature band at the PM, and the surface biotin labeling result verified that C538A indeed lost the PM localization (Figure 4C). And this mutant showed the loss of palmitoylation the same as the treatment of 2‐BP (Figure 4D). The N‐glycosylation/S‐palmitoylation combinatory mutant (N47Q/N151Q/C538A) was positioned as a bottom band the same as the removal of both N‐glycosylation and palmitoylation by tunicamycin treatment, which presumably represented the naïve Fzd5 without any modifications (Figure S4E, F, Supporting Information).

We then sought to understand the correlation of Fzd5 cholesterol binding with its palmitoylation. The cholesterol‐binding deficient mutants such as I199A showed a single immature band as C538A did (Figure S4E, Supporting Information). Combinatory mutation of I199A/C538A could not further down‐migrate the band position, as N47Q/N151Q/I199A positioned bottom the same as N47Q/N151Q/C538A. When we employed an APE assay to detect palmitoylation, we could not observe the band migration on cholesterol‐binding deficient mutants (Figure 4E). Cholesterol deprivation significantly reduced the palmitoylation of Fzd5, and the addition of cholesterol promoted the palmitoylation of Fzd5 WT but not C538A (Figure 4F). On the contrary, C538A maintained cholesterol binding capacity similar to WT and lost the binding when combined with I199A (Figure S4G, Supporting Information). To sum, these findings demonstrated that the palmitoylation at C538 is dependent on Fzd5 cholesterol binding, whereas the loss of palmitoylation does not affect the cholesterol binding.

We hypothesize that loss of cholesterol binding or S‐palmitoylation may cause the disruption of ER‐Golgi transportation of Fzd5 and thus constrains Fzd5 in the inner membrane. By employing fluorescent microscopic assay, we visualized that WT Fzd5 co‐localized well with ER, *cis*‐Golgi, and *tran*‐Golgi markers. When treated with statin or 2‐BP, Fzd5 showed significant reduction of co‐migration with *cis*‐Golgi and *trans*‐Golgi (Figure 4G–I). Similarly, either I199A or C538A showed low‐level of co‐existence with Golgi markers compared to WT (Figure S4H,I, Supporting Information). Since COPII complex mediates the ER‐Golgi transportation, we tested the co‐localization of Fzd5 with its core member Sec23B. WT Fzd5 co‐immunoprecipitated well with Sec23B as either I199A or C538A minimally interacted with Sec23B (Figure S4J, Supporting Information). Addition of cholesterol enhanced the interaction between Fzd5 and Sec23B whereas statin treatments reduced it (Figure S4K, Supporting Information). When transmembrane protein cannot be properly modified and transported to Golgi, it undergoes ER‐stress‐induced proteasomal degradation instead of lysosomal degradation. We observed that proteasome inhibitor MG132 significantly increased the protein amount of I199A and C538A instead of WT, as lysosome inhibitor Baf A1 only affected WT Fzd5 stability but I199A or C538A (Figure S4L, Supporting Information). Previously, Shisa and TMEM79 were reported to affect Fzd maturation and transportation to PM.^[^ ^6^, ^21^ ^]^ We showed that cholesterol modulation or I199A or C538A did not alter the affinity of Fzd5 with Shisa or TMEM79 (Figure S4M, N, Supporting Information). In terms of Fzd5‐mediated Wnt/*β*‐catenin signaling, C538A failed to respond to Wnt treatment and cholesterol changes in various assays such as luciferase assays and Western Blotting assay showing cytosolic *β*‐catenin, which was consistent with its ER trapping (Figure 4J–M; Figure S4O–R, Supporting Information). Taken together, these results indicate that palmitoylation at C538 is the major driving force for Fzd5 maturation and trafficking, which is dependent on cholesterol binding to the conserved residues at LS3. Loss of either cholesterol binding or the palmitoylation results in Fzd5 accumulation at ER and thus attenuates Wnt/*β*‐catenin signaling.

### 2.5 Cholesterol Modulates RNF43‐Mutant PDAC Growth via Fzd5‐Mediated Wnt/*β*‐Catenin Activity

**Figure 5:**
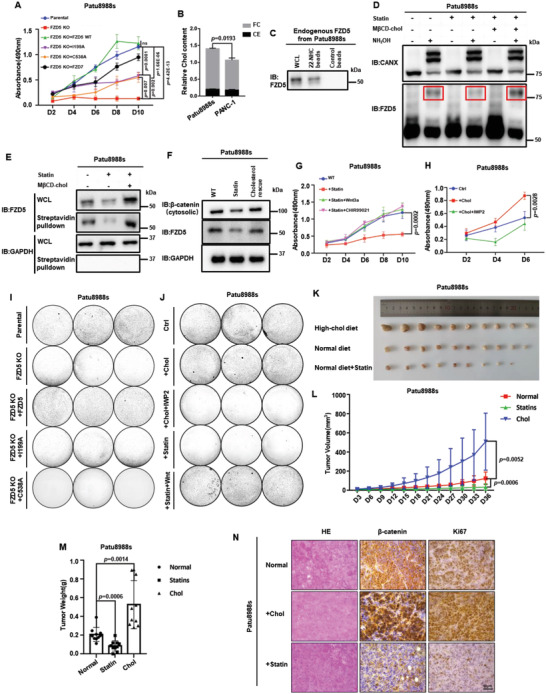
Cholesterol modulates RNF43‐mutant PDAC growth via Fzd5‐mediated Wnt/*β*‐catenin activity. A) MTT cell growth assay for Patu8988s Fzd5‐KO cell line and rescues. Error bars mean ± SD, n = 3 replicates, by two‐way ANOVA analysis. B) Quantities of cholesterol contents in PDAC cells. FC: free cholesterol; CE: cholesterol ester. Error bars mean ± SD, n = 3 replicates, by two‐tailed unpaired student's t‐test analysis. C) 22‐NHC pulldown assay of endogenous Fzd5 extracted from Patu8988s cells. D) APE‐assay of endogenous Fzd5 in Patu8988s cells upon cholesterol starvation and repletion treatments. Stripes in the red boxes present palmitoylated Fzd5. CANX is used as control. E) Surface biotin labeling assay of endogenous Fzd5 upon cholesterol starvation and repletion treatments. F) Cytosolic *β*‐catenin assay of Patu8988s cell under normal, statin treatment and cholesterol rescue conditions. G) MTT cell growth assay for Patu8988s cells under normal, statin, statin+Wnt3a, and statin+CHIR99021 treatment conditions. Error bars mean ± SD, n = 3 replicates, by two‐way ANOVA analysis. H) MTT cell growth assay for Patu8988s cell under normal, cholesterol addition, cholesterol addition+IWP2 (Porcupine inhibitor) conditions. Error bars mean ± SD, n = 3 replicates, by two‐way ANOVA analysis. I) Colony formation assay of parental, Fzd5‐KO and rescued Patu8988s cells. Each set was triplicated. J) Colony formation assay of Patu8988s cell under normal, additional cholesterol, additional cholesterol+IWP2, statin, statin+Wnt treatment conditions. Each set was triplicated. K) The PDAC tumors formed by subcutaneous implantations of Patu8988s cells. From top to bottom, the rows represent high‐cholesterol diet fed group, normal diet fed group, and normal diet plus pravastatin fed group, respectively. n = 10 for each group. L) Subcutaneously implanted tumor size measurement by days. Error bars mean ± SD, n = 10 for each group, by two‐way ANOVA analysis. M) Subcutaneously implanted tumor weight measurements after section at Day36. Error bars mean ± SD, n = 10 for each group, by one‐way ANOVA analysis. N) Immunohistochemistry of subcutaneously implanted tumors and hematoxylin‐eosin staining. For all MTT experiments, D: day. 6‐well plates are used for all colony formation assays. Each circle represents a whole well of a 6‐well plate. ns: not significant.

As a special feature, PDAC generally requires highly active cholesterol metabolism to survive and grow, though the in‐depth mechanistic understanding remains elusive.^[^ ^14a,d^ ^]^ In RNF43‐mutant PDAC, Fzd5‐mediated Wnt/*β*‐catenin activity is required for tumor survival and growth.^[^ ^5b^ ^]^ We thus proposed that Fzd5 may act as a critical “bridge” to couple aberrant cholesterol metabolism with Wnt signaling in RNF43‐mutant PDAC. We first employed CRISPR‐Cas9 system to knockout Fzd5 in two RNF43‐mutant cell lines (HPAF‐II and Patu8988s) and two RNF43‐WT cell lines (BxPC3 and PANC‐1) to observe the cell growth (Figure S5A, B, Supporting Information). As expected, RNF43‐mutant PDAC showed significant growth retardation after Fzd5 KO, as exogenous Wnt rescued the growth (Figure S5C, Supporting Information). Re‐expression of either WT Fzd5 or Fzd7 can rescue the tumor growth but Fzd5 I199A or C538A (**Figure** **5**A; Figure S5D, Supporting Information). Next, we examined the cholesterol level in PDAC. RNF43‐mutant PDAC cell contains higher cholesterol contents than RNF43‐WT PDAC (Figure 5B). This difference in cholesterol homeostasis was also supported by TCGA (The Cancer Genome Atlas) database, which showed the differentially expressed genes (DEGs) between RNF43‐mutant and ‐WT PDAC significantly enriched in sterol/lipid metabolism and transport processes (Figure S5E, F, Supporting Information). Further, we collected twelve primary PDAC tumor samples from patients, and identified two of these cases being RNF43‐mutant by whole‐exome sequencing (Table S1, Supporting Information). Then, we performed lipidomic study (untargeted liposome analysis) on these samples to identify the cholesterol‐related lipid metabolites (Table S2, Supporting Information). As shown by the heatmap, RNF43‐mutant PDAC tumor samples had a distinct cholesterol metabolic pattern as compared to RNF43‐WT samples (Figure S5G, Supporting Information). In particular, the content of total cholesteryl esters (CEs) in RNF43‐mutant samples was threefold higher than that in RNF43‐WT samples with strong statistical significance, as other unesterified sterol contents also showed alterations (Figure S5H–J, Supporting Information). It was previously known that excessive free cholesterol may cause cytotoxic effects including membrane rigidity change and intracellular structure disruption.^[^ ^22^ ^]^ Being a common approach, cholesterol storage as CE in cells can prevent that effect, as disruption of which causes cell death.^[^ ^9^, ^14^ ^]^ Since cells can dynamically sense and convert different sterol contents, the large quantity of CE in RNF43‐mutant PDAC can serve as a reservoir to readily secure a relatively high cellular cholesterol level. Therefore, we have confirmed Fzd5 dependence and the aberrant cholesterol metabolism in RNF43‐mutant PDAC. And we argue that the prolific cholesterol pool in RNF43‐mutant PDAC sustains continuous Fzd5 maturation and Wnt/*β*‐catenin‐dependent cancer growth.

We then experimentally studied the correlation of cholesterol level with Fzd5‐mediated Wnt/*β*‐catenin signaling. We first proved that endogenous Fzd5 could specifically bind to 22‐NHC beads (Figure 5C). Like the ectopically expressed Fzd5, the palmitoylation of the endogenous Fzd5 was also affected by the cellular cholesterol starvation and repletion treatment (Figure 5D). Meanwhile, by surface biotin labeling and cellular fractionation assays, it was shown that cholesterol starvation reduced the PM‐localized endogenous Fzd5, as conversely, cholesterol addition stimulated PM residence of endogenous Fzd5 (Figure 5E; Figure S6A, Supporting Information). Similar to exogenous Fzd5, the endogenous Fzd5 level could be elevated in the presence of lysosomal inhibitor Baf A1 but proteasomal inhibitor MG132 (Figure S6B, Supporting Information). But upon statin treatment, Baf A1 failed to revert the diminishment of the endogenous Fzd5, thus indicating that cholesterol starvation did not induce Fzd5 ubiquitin‐lysosomal degradation (Figure S6B, Supporting Information). In contrast, MG132 treatment resulted in the accumulation of endogenous Fzd5 only in the presence of statin, which suggested that endogenous Fzd5 accumulated in the inner membranes and underwent ER‐stress‐induced proteasome degradation during cholesterol starvation (Figure S6B, Supporting Information). These results were similar to those of ectopically expressed Fzd5 in Figure S4L, Supporting Information. To strengthen this notion, we also showed that treatment of Rspo1 or knockdown of ZNRF3 in RNF43‐mutant PDAC could not rescue the effect of statin treatment (Figure S6C–E, Supporting Information), which further excluded the possibility that cholesterol affects endogenous Fzd5 by ubiquitination and sequel lysosomal degradation. Therefore, we conclude that the endogenous Fzd5 behaved similarly to the ectopically‐expressed Fzd5 in sensing cellular cholesterol. Cholesterol level positively correlates with the endogenous Fzd5 palmitoylation and PM localization. And the reduction of endogenous Fzd5 upon cholesterol starvation is independent of Fzd5 ubiquitin‐lysosomal degradation.

Functionally, we observed that endogenous Fzd5 responded to cholesterol level change in the same way as exogenous Fzd5, and it positively correlated with cytosolic *β*‐catenin accumulation and Wnt target gene expression (Figure 5F; Figure S6F, Supporting Information). Cholesterol starvation by statin significantly slowed down the growth of RNF43‐mutant PDAC, which could be rescued by additional exogenous Wnt or GSK3 inhibitor (Figure 5G). Cholesterol addition in the culture medium, on the other hand, stimulated RNF43‐mutant PDAC growth, and this effect could be reverted by Porcupine inhibitor IWP‐2 (Figure 5H). As expected, in Fzd5 KO PDAC, rescuing by Fzd5 I199A, C538A or Fzd7 did not respond to faster cell growth cholesterol‐dependently (Figure S6G, Supporting Information). In clonogenic growth assays, we also found that Fzd5 WT but I199A or C538A could rescue the growth of Fzd5 KO Patu8988s/HPAF‐II cells (Figure 5I; Figure S6H, Supporting Information). Higher cholesterol levels enhanced the colony formation of RNF43‐mutant PDAC in the absence of IWP2, and cholesterol depletion slowed down the colony formation which could be rescued by exogenous Wnt (Figure 5J). For in vivo study, we subcutaneously transplanted RNF43‐mutant PDAC onto nude mice. Feeding the mice with high cholesterol diet significantly increased the tumor growth, as treating the mice with statin showed the opposite effect (Figure 5K–M). And consistent with the in vitro results, the proportion of endogenous Fzd5 that underwent S‐palmitoylation significantly increased in the high cholesterol diet group and shrank in the statin‐treated group (Figure S6I, Supporting Information). Notably, the tumor growth of Fzd7‐rescued Fzd5‐KO Patu8988s did not alter by cholesterol modulation (Figure S6J–L, Supporting Information). In immunohistochemistry assay, it was clearly seen that high‐cholesterol diet‐fed mice showed strong staining for proliferation and *β*‐catenin in parental PDAC but not Fzd7‐rescued PDAC (Figure 5N; Figure S6M, Supporting Information). Together, these results suggest that the growth of the RNF43‐mutant PDAC is sensitive to the cellular cholesterol content. As the upstream event, aberrant cholesterol metabolism governs RNF43‐mutant PDAC growth specifically through Fzd5‐mediated Wnt/*β*‐catenin signaling.

### 2.6 25‐Hydroxysterol Alleviates PDAC Tumor Burden by Inhibiting Cholesterol‐Fzd5‐Wnt/*β*‐Catenin Axis

**Figure 6:**
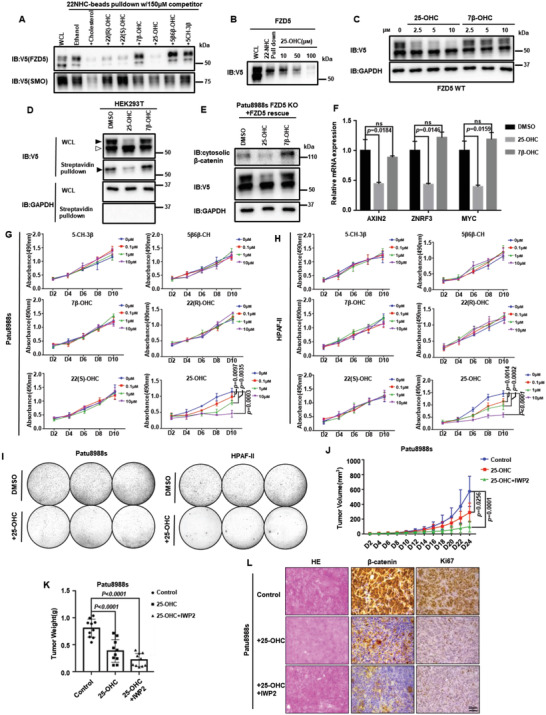
25‐hydroxysterol alleviates PDAC tumor burden by inhibiting cholesterol‐Fzd5‐Wnt/*β*‐catenin axis. A) 22‐NHC beads pulldown and competition assay. Ectopically expressed V5‐tagged Fzd5 or Smo was pulled down by 22‐NHC and competed by various oxysterols. B) 22‐NHC beads pulldown and competition assay of V5‐tagged Fzd5 by different doses of 25‐OHC. C) WB assay showing 25‐OHC inhibits Fzd5 maturation at different doses. D) Surface biotin labeling assay of Fzd5 under oxysterol treatments. E) WB assay showing 25‐OHC reduces cytosolic *β*‐catenin level in Patu8988s cells. F) RT‐qPCR assay showing treatment of 25‐OHC inhibited Wnt target genes including AXIN2, ZNRF3, and MYC in Patu8988s cells. Error bars mean ± SD, n = 3 replicates, by one‐way ANOVA analysis. G,H). MTT cell growth assay of Patu8988s (G) and HPAF‐II (H) under various oxysterol treatments. Error bars mean ± SD, n = 3 replicates, by two‐way ANOVA analysis (If the p‐value is not specified, it is greater than 0.05). I) Colony formation assay of Patu8988s and HPAF‐II cells upon 25‐OHC treatment. Each set was triplicated. J) Subcutaneously implanted tumor size measurements by days of control group, 25‐OHC treatment group, and 25‐OHC+IWP2 treatment group. Error bars mean ± SD, n = 10 for each group, by two‐way ANOVA analysis. K) Subcutaneously implanted tumor weight measurements after section on Day24. Error bars mean ± SD, n = 10 for each group, by one‐way ANOVA analysis. L) Immunohistochemistry of subcutaneously implanted tumors and hematoxylin‐eosin staining. For all MTT experiments, D represents day. 6‐well plates are used for colony formation assay. Each circle represents a whole well of a 6‐well plate. ns: not significant.

We next sought to use oxysterols to outcompete cholesterol and retain Fzd5 at the inner membrane like the inhibition of 22‐NHC to Smo for potential therapeutic applications.^[^ ^10a^ ^]^ We thus screened several common cholesterol derivatives by competition binding assays. The result clearly showed that among these derivatives, 25‐OHC could effectively outcompete cholesterol in terms of Fzd5 binding in a dose‐dependent manner for both V5‐tagged Fzd5 expressed in HEK293T cells and the endogenous Fzd5 extracted from Patu8988s cells (**Figure** **6**A,B; Figure S7A,B, Supporting Information). Functionally, the addition of 25‐OHC to cell culture significantly reduced the matured band of Fzd5 and prevented Fzd5 from PM localization (Figure 6C, D; Figure S7C, Supporting Information).

In RNF43‐mutant PDAC, 25‐OHC could also effectively reduce the Fzd5‐dependent Wnt/*β*‐catenin activity (Figure 6E, F). And only 25‐OHC could inhibit the RNF43‐mutant PDAC growth dose‐dependently in both 2D and 3D, though other oxysterols could also modulate cholesterol metabolism gene expressions (Figure 6G–I; Figure S7D, Supporting Information). Eventually, we tested the tumor‐suppressive effect of 25‐OHC in vivo. The size and weight of the subcutaneous implanted PDAC reduced significantly under 25‐OHC treatment, and the combinatory treatment with IWP‐2 showed a more drastic effect in tumor clearance (Figure 6J–L; Figure S7E, Supporting Information). To conclude, these results demonstrated that 25‐OHC outcompetes cholesterol and constrains Fzd5 at the inner membrane. 25‐OHC treatment lessons Fzd5‐dependent Wnt/*β*‐catenin signaling and causes the mitigation of the tumor burden of RNF43‐mutant PDAC.

## 3 Discussion

**Figure 7:**
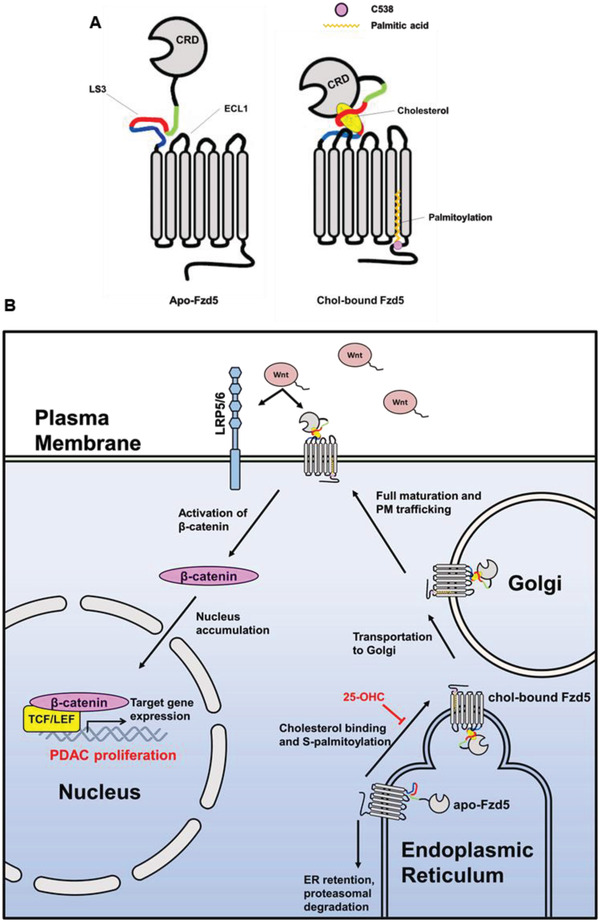
Working model of cholesterol affecting RNF43‐mutant PDAC growth through Fzd5‐mediated Wnt/*β*‐catenin signaling. A) Cholesterol binding to Fzd5 enables the conserved C538 to be palmitoylated. B) Cholesterol‐coupled palmitoylation allows Fzd5 for ER exit and maturation in Golgi, thus potentiates RNF43‐mutant PDAC growth through Fzd5‐mediated Wnt/*β*‐catenin signaling. 25‐OHC competes with cholesterol and inhibits Fzd5 maturation.

As a crucial receptor that regulates multiple biological processes through Wnt signaling, understanding the biogenesis anof Fzd has been a major goal in the past decade. Also, researchers started to realize that different Fzd subtypes have distinct functions and regulations.^[^ ^3^, ^5^, ^7^, ^23^ ^]^ Thus, understanding the difference and redundancy of 10 Fzd subtypes have been appealing but challenging work. For the first time, we discovered that cholesterol specifically binds to a particular Fzd subtype, Fzd5. And the binding to cholesterol enabled Fzd5 maturation and PM localization. Because of the structural difference between Fzd and Smo CRD, it was suggested that Fzd is unable to bind cholesterol. When we sought the potential cholesterol binding site, we surprisingly found that the conserved residues at the extracellular linker/loop region are essentially required (**Figure** **7**A). Apparently, this binding mechanism is distinct from Smo‐cholesterol or Fzd1/2/7‐fatty acid interaction which mainly requires CRD. Though one structural study suggested that the extracellular loops are directly involved in cholesterol binding, the authors would still consent that Smo CRD‐sterol interaction is critical.^[^ ^10f,g^ ^]^ We hypothesize that cholesterol binding enables Fzd5 to a certain conformation that allows its maturation and function, which was also seen in other transmembrane receptors.^[^ ^10^, ^24^ ^]^ This study thus provided another example of cholesterol being a signaling molecule regulating protein conformation and activity.

As mentioned above, cholesterol and fatty acids were reported to promote PM localization of Smo and Fzd1/2/7, respectively. However, how lipid binding allowed these processes molecularly remained mysterious. S‐palmitoylation on transmembrane protein has been reported to play a pivotal role in protein maturation and trafficking.^[^ ^19b^ ^]^ For instance, the S‐palmitoylation promotes the ER exit of LRP6 and ABCA1.^[^ ^19a,c^ ^]^ But this type of post‐translational modification was not reported in any Fzd class receptor. In this study, we identified the S‐palmitoylation site of Fzd5 within its cytoplasmic tail for the first time (Figure 7A). And we indicated that this S‐palmitoylation is required for Fzd5 ER‐Golgi transportation for further maturation and receptor function. Logically, we argued that the S‐palmitoylation is the consequence of cholesterol binding. Loss of cholesterol binding resulted in the S‐palmitoylation deficiency and the ER retention of Fzd5 for proteasome‐dependent protein degradation. We hypothesize that the cholesterol binding enables Fzd5 conformational change and allosterically recruits acyltransferase in ER to recognize the conserved cytoplasmic cysteine.

In this work, we correlated Fzd5‐mediated Wnt/*β*‐catenin signaling with cholesterol metabolism in RNF43‐mutant PDAC (Figure 7B). We found that additional cholesterol promoted Fzd5‐mediated Wnt/*β*‐catenin signaling and PDAC growth both in vitro and in vivo. Although cholesterol plays as a basic building block and energy source, we argue that in RNF43‐mutant PDAC cholesterol mainly functions through modulating Fzd5 maturation and trafficking, thus sustaining sufficient Wnt/*β*‐catenin activity for its survival and growth. Based on the Wnt‐addicted nature of RNF43‐mutant PDAC, Porcupine inhibitor has been proved effective to suppress its growth.^[^ ^13^, ^25^ ^]^ We proposed that certain cholesterol derivatives such as oxysterol, may both block Fzd5‐mediated Wnt/*β*‐catenin signaling and de novo cholesterol synthesis. Eventually, we identified a natural oxysterol, 25‐OHC, could effectively cause the retention of Fzd5 at the inner membranes thus exerting a suppressive effect on both Wnt/*β*‐catenin signaling and cholesterol metabolism. And the growth of RNF43‐mutant PDAC can be effectively inhibited by 25‐OHC dose‐dependently. This finding has thus provided a new strategy for treating Wnt‐driven cancers.

Despite the breakthrough, further answers to many aspects regarding this novel finding need to be addressed in future research. The first major question is how exactly cholesterol binds to Fzd5. However, until the full‐length Fzd5‐cholesterol co‐structure is solved, this question would remain uncertain. The second question would be how cholesterol binding could affect its S‐palmitoylation. In our hypothesis, the binding to cholesterol recruits certain acyltransferase or exposes the cysteine by conformational change, which requires structural study to fully elucidate. In the most recent attempt to solve Fzd5 structure, neither the complete extracellular linker region nor the cysteine in cytoplasmic tail is resolved.^[^ ^3b^ ^]^ We expect to discover the acyltransferase that palmitoylates Fzd5 in our follow‐up study and hopes that the high‐resolution Fzd5 structure to be solved by cryo‐electron microscopy in the near future.

## 4 Experimental Section

### Reagents and Resources Table

**Table:** 

Reagent or Resource	Source	Identifier
Antibodies		
V5‐Tag (D3H8Q) Rabbit mAb	Cell Signaling Technology	#13202
HA‐Tag (C29F4) Rabbit mAb	Cell Signaling Technology	#3724
Myc‐Tag (9B11) Mouse mAb	Cell Signaling Technology	#2276
DYKDDDDK Tag (D6W5B) Rabbit mAb	Cell Signaling Technology	#14793
E‐Cadherin (24E10) Rabbit mAb	Cell Signaling Technology	#3195
*β*‐Catenin (D10A8) XP Rabbit mAb	Cell Signaling Technology	#8480
LRP6 (C5C7) Rabbit mAb	Cell Signaling Technology	#2560
Phospho‐LRP6 (Ser1490) Antibody	Cell Signaling Technology	#2568
Ki‐67 (8D5) Mouse mAb	Cell Signaling Technology	#9499
Anti‐biotin, HRP‐linked Antibody	Cell Signaling Technology	#7075
Anti‐Frizzled 5 Rb Polyclonal Antibody	Sigma‐Aldrich	06‐756
Wnt3a (C64F2) Rabbit mAb	Cell Signaling Technology	#2721
Calnexin Rb Polyclonal antibody	Proteintech	10427‐2‐AP
GOLGA2/GM130 Rb Polyclonal antibody	Proteintech	11308‐1‐AP
Syntaxin 6 Rb Polyclonal antibody	Proteintech	10841‐1‐AP
Anti‐LDLR Mouse monoclonal Antibody (C7)	Santa Cruz Biotechnology	SC‐18823
Anti‐mouse IgG, HRP‐linked Antibody	Cell Signaling Technology	#7076
Anti‐rabbit IgG, HRP‐linked Antibody	Cell Signaling Technology	#7074

**Table:** 

Reagent or Resource	Source	Identifier
Goat anti‐Rabbit IgG (H+L) Highly Cross‐Adsorbed Secondary Antibody, Alexa Fluor 555	Invitrogen	A21429
Bacterial strand		
*E. coli* NEB 5‐alpha	New England Biolabs (NEB)	C2987H
Chemicals		
Cholesterol	Sigma‐Aldrich	C8667
Pregnenolone	Aladdin	P129412
4,7,10‐trioxa‐1,13‐tridecanediamine	Sigma‐Aldrich	369519
NaBH(OAc)_3_	Sigma‐Aldrich	316393
1,2‐dichloroethane	Sigma‐Aldrich	284505
Triethylamine	Sigma‐Aldrich	471283
Ethanolamine	Sigma‐Aldrich	E9508
Pravastatin sodium	Sigma‐Aldrich	P4498
Methyl‐*β*‐cyclodextrin	Sigma‐Aldrich	332615
Sulfo‐NHS‐SS‐Biotin	APExBIO	A8005
SNAP‐Surface 549	New England Biolabs (NEB)	#S9112S
SNAP‐Cell Oregon Green	New England Biolabs (NEB)	#S9104S
Cycloheximide	Sigma‐Aldrich	5087390001
2‐Bromohexadecanoic acid	Sigma‐Aldrich	21604
Triethanolamine	Sigma‐Aldrich	V900257
Methoxypolyethylene glycol maleimide	Sigma‐Aldrich	712469
Hydroxylamine	Sigma‐Aldrich	438227
N‐Ethylmaleimide	Sigma‐Aldrich	E3876
Tris(2‐carboxyethyl) phosphine hydrochloride	Sigma‐Aldrich	C4706
IWP‐2	Sigma‐Aldrich	I0536
CHIR99021	Cayman	13122
Tunicamycin	Aladdin	T101151
7*β*‐Hydroxycholesterol	Aladdin	C130187
25‐Hydroxycholesterol	Aladdin	C130176
22(R)‐Hydroxycholesterol	Sigma‐Aldrich	H9384
5‐Cholesten‐3*β*‐ol‐7‐one	Sigma‐Aldrich	C2394
22(S)‐Hydroxycholesterol	Sigma‐Aldrich	H5884
Cholesterol 5*β*,6*β*‐epoxide	Sigma‐Aldrich	C2648
Thiazolyl Blue Tetrazolium Bromide	Sigma‐Aldrich	M5655
Hoechst	Thermo	1990363
Puromycin	Invivogen	ant‐pr‐1
Geneticin	Invivogen	ant‐gn‐1

**Table:** 

Reagent or Resource	Source	Identifier
Gibco Penicillin‐Streptomycin‐Glutamine (100X)	Gibco	10378016
Polybrene	Santa Cruz Biotechnology	SC‐134220
Lipoprotein Deficient Serum	Sigma‐Aldrich	S5519
Bafilomycin A1	Santa Cruz Biotechnology	SAC‐201550A
MG132	Beyotime Biotechnology	S1748
Poly‐L‐lysine	Sango Biotech	A600751
Citrate Antigen Retrieval Solution	Sango Biotech	E673002
Crystal violet	Sango Biotech	A100528
Lipofectamine 2000 Transfection Reagent	Invitrogen	11668019
Ampicillin	Sango Biotech	A100339
4% paraformaldehyde	Beyotime Biotechnology	P0099
Alk‐16(Palmitic acid (15‐yne))	Sigma‐Aldrich	900400P
Biotin‐azide	APExBIO	A8013
Tris[(1‐benzyl‐1H‐1,2,3‐triazol‐4‐yl) methyl] amine	Sigma‐Aldrich	678937
Digitonin	Sigma‐Aldrich	D141
Complete Protease Inhibitor Cocktail	Roche	04693116001
Triton X‐100	Sigma‐Aldrich	X100
Critical commercial assays/kits		
Dual Luciferase Reporter Gene Assay Kit	Beyotime Biotechnology	RG027
BCA Protein Assay Kit	Thermo	23227
Endo‐free Plasmid Mini Kit	Omega	D6950
Minute Plasma Membrane Protein Isolation and Cell Fractionation Kit	Inventbiotech	SM‐005
UNlQ‐10 Column Trizol Total RNA Isolation Kit	Sango Biotech	B511321
MonScript RTIII All‐in‐One Mix with dsDNase	Monad	MR05101M
MonAmp ChemoHS qPCR Mix	Monad	MQ00401S
GeneJET Gel Extraction Kit	Thermo	K0692
GeneJET PCR Purification Kit	Thermo	K0702
UltraSensitiveTM SP (Mouse/Rabbit) IHC Kit	MXB Biotechnologies	KIT‐9720
DAB Kit (20×)	MXB Biotechnologies	DAB‐0031

**Table:** 

Reagent or Resource	Source	Identifier
Phanta Max Super‐Fidelity DNA Polymerase	Vazyme	P505‐d1
Hematoxylin‐Eosin (HE) staining kit	BBI LIFE SCIENCES CORPORATION	E607318
Chemistar High‐sig ECL Western Blotting Substrate	Tanon	180‐5001
Experimental models: Cell lines		
HEK293T	ATCC	CRL‐11268 RRID:CVCL_1926
HEK293T BAR/Renilla (B/R)	This study	
HEK293T Z/R DKO	Gifted by Feng Cong PMID: 25891077	
HEK293T FZD‐null	Gifted by Feng Cong PMID: 32601235	
Patu8988s	Procell	CL‐0303
HPAF‐II	NCACC, China	SCSP‐5011
BxPC3	NCACC, China	TCHu 12
PANC‐1	NCACC, China	SCSP‐ 535
L cell	ATCC	CRL‐2648
L‐Wnt3a cell	ATCC	CRL‐2647
Experimental models: Animals		
BALB/cA‐Nude mice	Beijing HFK Bioscience CO., LTD	13001A
Oligonucleotides		
Fzd5 sgRNA sequence	PMID: 27869803	CAAGGTCCATTTCTTGGCTGT
#1‐shLDLR sequence	This study	ATATCTTCGCATCTTCGCTGG
#2‐shLDLR sequence	This study	TACTTTGTCCTCAAAGACGGC
Control‐shRNA sequence	This study	AAAAAAAAAAAAAAAAAAAAA
#1‐shZNRF3 sequence	This study	GCTGCTACACTGAGGACTATT
#2‐shZNRF3 sequence	This study	CACTGGGCCTATGTAATAATT
FZD5‐RT‐qPCR primers	This study	F:CCGTTCGTGTGCAAGTGTC R: GAAGCGTTCCATGTCGATGAG
CTNNB1‐RT‐qPCR primers	This study	F: AAAGCGGCTGTTAGTCACTGG R: CGAGTCATTGCATACTGTCCAT
AXIN2‐RT‐qPCR primers	This study	F: AGCCAAAGCGATCTACAAAAGG R:AAGTCAAAAACATCTGGTAGGCA
MYC‐RT‐qPCR primers	This study	F: GTCAAGAGGCGAACACACAAC R: TTGGACGGACAGGATGTATGC
ZNRF3‐RT‐qPCR primers	This study	F: GGGTCATCCCCTGTACTCAC R: TTGTCCTCGTAGGGTAGGCTG
LDLR‐RT‐qPCR primers	This study	F: ACGGCGTCTCTTCCTATGACA R: CCCTTGGTATCCGCAACAGA
ABCA1‐RT‐qPCR primers	This study	F: TTCCCGCATTATCTGGAAAGC R: CAAGGTCCATTTCTTGGCTGT

**Table:** 

Reagent or Resource	Source	Identifier
GAPDH‐RT‐qPCR primers	This study	F: GGAGCGAGATCCCTCCAAAAT R: GGCTGTTGTCATACTTCTCATGG
Recombinant DNA		
Lenti‐EF1*α*‐puro V5‐Fzd1	This study	
Lenti‐EF1*α*‐puro V5‐Fzd2	This study	
Lenti‐EF1*α*‐puro V5‐Fzd3	This study	
Lenti‐EF1*α*‐puro V5‐Fzd4	This study	
Lenti‐EF1*α*‐puro V5‐Fzd5	This study	
Lenti‐EF1*α*‐puro V5‐Fzd6	This study	
Lenti‐EF1*α*‐puro V5‐Fzd7	This study	
Lenti‐EF1*α*‐puro V5‐Fzd8	This study	
Lenti‐EF1*α*‐puro V5‐Fzd9	This study	
Lenti‐EF1*α*‐puro V5‐Fzd10	This study	
Lenti‐EF1*α*‐puro V5‐Smo	This study	
Lenti‐EF1*α*‐puro V5‐SNAP‐Fzd5	This study	
Lenti‐EF1*α*‐puro V5‐SNAP‐Fzd7	This study	
Lenti‐EF1*α*‐puro HA‐Tmem79	This study	
Lenti‐EF1*α*‐puro HA‐Shisa3	This study	
Lenti‐EF1*α*‐puro Flag‐Sec23B	This study	
Lenti‐EF1*α*‐puro His6‐V5‐Fzd5	This study	
Lenti‐EF1*α*‐puro His6‐V5‐Fzd7	This study	
Lenti‐EF1*α*‐puro Flag‐LDLR	This study	
pCMV‐VSV‐G	Addgene	#8454
psPAX2	Addgene	#12260
pLKO‐puro shLDLR	This study	
Lenti‐Cas9‐puro sgFZD5	This study	
pLKO‐puro shZNRF3	This study	
pCS2	This study	
Software and algorithms		
Graphpad Prism 8	Graphpad software	https://www.graphpad.com
ImageJ	^[^ ^26^ ^]^	https://imagej.nih.gov/ij/
PyMol Educational	PyMOL by Schrödinger	https://pymol.org/edu/?q=educational/
PhotoShop CC2019	Adobe	https://www.adobe.com/cn/products/photoshop.html
Origin 2019	OriginLab Corporation	https://www.originlab.com
Other		
*α*‐V5 Agarose Affinity Gel	Sigma‐Aldrich	A7345
Affigel‐10 beads	Bio‐Rad	#1085
Pierce Streptavidin Agarose	Pierce	20349
*α*‐HA Agarose Affinity Gel	Sigma‐Aldrich	A2095
*α*‐Flag Agarose Affinity Gel	Sigma‐Aldrich	4596
Ni‐NTA resin	Qianchunbio	A41002‐05

### Clinical Samples

Surgical specimens of pancreatic cancers were obtained from 12 patients, all resected from January 2017 to December 2020 from the Tongji Hospital, Tongji Medical College, Huazhong University of Science and Technology (Wuhan, China). All cancers were verified as adenocarcinomas. No patients received preoperative chemotherapy or radiotherapy. The use of clinical samples was approved by the Human Research Ethics Committee of the Tongji Hospital, Tongji Medical College, Huazhong University of Science and Technology (Wuhan, China) (protocol No.TJ‐IRB20190418). Written consent from all participants or next of kin was obtained prior to the research.

### Animal Work

Animals were purchased from Beijing HFK Bioscience CO., LTD. Mice maintenance and treatments described were approved by the Research Ethics Committees of the College of Life and Health Sciences of Northeastern University (Approval no. 16099 M).

### High‐Cholesterol Diet and Statin Treatment

Six weeks old nude mice (BALB/cA) (Beijing HFK Bioscience CO., LTD) were separately treated in a group of 10 by either normal diet (Beijing HFK Bioscience CO., LTD), high cholesterol diet (HCD) containing 2% cholesterol and 0.25% sodium cholate) (Beijing HFK Bioscience CO., LTD) or normal diet containing pravastatin sodium (40mg kg^−1^ day^−1^,Sigma). Experiments were performed using these mice.

### Subcutaneous Tumor Transplantation

Patu8988s cells were trypsinized, washed, and resuspended in PBS. Designed cell number and viability were determined using trypan blue. Nude mice (Beijing HFK Bioscience CO., LTD) were subcutaneously injected with Patu8988s cells at a density of 1× 10^6^ cells per site.

### 25‐OHC and IWP‐2 Treatment

Thirty 6‐week old nude mice (Beijing HFK Bioscience CO., LTD) were randomly divided into three groups (10 mice/group) followed by subcutaneous injection with Patu8988s cells at a density of 1× 10^6^ cells per site. Then the first group was intraperitoneally injected with 10 µL PBS every 2 days. The second group was intraperitoneally injected with 2mg kg^−1^ 25‐OHC (aladdin) every 2 days. The third group was intraperitoneally injected with 2mg kg^−1^ 25‐OHC (aladdin) and 20 µm IWP‐2 (Sigma) every 2 days.

### Cell Lines

This study utilized HEK293T (parental and genetically modified), Patu8988s, HPAF‐II, BxPC3, PANC‐1, L, and L‐Wnt3a cells. All cell lines were maintained in humidified incubators with 5% CO_2_ at 37°C. HEK293T (parental and genetically modified), PANC‐1, L, and L‐Wnt3a cells were cultured in DMEM‐High Glucose supplemented with 10% FBS (fetal bovine serum) and 100 mg mL^−1^ of penicillin/streptomycin/glutamine (Gibco). Patu8988s and BxPC3 were cultured in RPMI‐1640 medium supplemented with 10% FBS and 100 mg mL^−1^ of penicillin/streptomycin/glutamine. HPAF‐II cells were cultured in DMEM Medium supplemented with 10% FBS, 1% Non‐Essential Amino Acids (Solarbio), and 100 mg mL^−1^ of penicillin/streptomycin/glutamine.

### Clones and Constructs

The plasmids expressing V5‐tagged full‐length Fzd1‐10, all V5‐tagged Fzd5 mutants, HA‐tagged Shisa3, HA‐tagged TMEM79, and Flag‐tagged LDLR were all constructed in the customized Lenti‐EF1*α*‐puro vector (primer sequence in the reagent). The plasmid containing shLDLR and shZNRF3 were constructed in pLKO vector (sequence in the reagent). The plasmid containing sgFzd5 was constructed in Lenti‐Cas9‐puro vector (sequence in the reagent). All plasmids were transformed in *E. coli* NEB 5‐alpha for amplification and extracted by OMEGA Endo‐free Plasmid Mini Kit. The concentrations of all plasmids were determined by Thermo Nanodrop 2000.

### Cell Culture and Transfection

Patu8988s cell was purchased at Procell. HPAF‐II, PANC‐1 and BxPC‐3 cells were purchased from the NCACC, China. HEK293T, L, and L‐Wnt3a cells were purchased from ATCC. FZD‐null HEK293T cell and Z/R DKO HEK293T cell were gifted from Dr. Feng Cong from Novartis. HEK293T (parental and genetically modified), PANC‐1, L, and L‐Wnt3a Cells were grown in DMEM Medium supplemented with 10% FBS. Patu8988s and BxPC‐3 cells were grown in RPMI 1640 medium with 10% FBS. HPAF‐II was grown in DMEM Medium supplemented with 10% FBS and 1% Non‐Essential Amino Acids (Solarbio). Exogenous Wnt3a was added for Fzd5 KO Patu8988s and HPAF‐II cells for cell maintenance and passaging but not in any experiment unless particularly mentioned.

Transfection was done using Invitrogen Lipofectamine 2000. After dilution of plasmids and Lipofectamine 2000 by using DMEM, plasmids were mixed with Lipofectamine 2000. The complex was incubated for 20 min at room temperature (RT), and added to HEK293T (parental or modified) cells in a growth medium. After 24–48 h, cells were lysed using Passive Lysis Buffer (25 mm Tris‐HCl, 150 mm NaCl, 1% NP40).

### Lentivirus Production and Infection

For lentivirus production, psPAX2 vector (for packaging, Addgene) and pCMV‐VSV‐G (for enveloping, Addgene) and desired customized Lenti‐EF1*α*‐puro plasmids, Lenti‐Cas9‐puro sgFZD5, or other lentiviral vector‐based plasmids were co‐transfected in HEK293T cell by the ratio of 5:1:5 in mass (ng). After transfection for 24 h, the medium was replaced by a fresh medium to produce virus‐containing conditioned medium. The virus‐containing conditioned medium was collected after 72 h and followed by concentration if necessary. For lentiviral infection, the cell culture was added to 0.5–1 mL virus‐containing conditioned medium or 0.1–0.5 mL virus concentrate for 48 h. After 48 h, the culture was refreshed and a certain selection drug was added for 0resistance selection.

### Cell Line Generation

HEK293T BAR/Renilla (B/R) cell line and HEK293T Fzd‐null BAR/Renilla (B/R) cell line: Lentivirus containing 7TFP and Renilla were generated as described above. HEK293T cell or HEK293T Fzd‐null cell were infected by the abovementioned lentivirus for 72 h. After that, the medium was removed and replenished with fresh medium containing puromycin (Invivogen) for selection. The selection was finished after 72 h. Eventually, the selected cells were cultured in normal medium and validated by dual‐luciferase reporter assay. Fzd5 KO cell lines: The selected sgRNA sequence (AGCAGCACTACCGCGAGAGC) were cloned into Lenti‐Cas9‐puro vectors (Addgene). Patu8988s, HPAF‐II, PANC‐1, and BxPC‐3 cells stably expressing Lenti‐Cas9‐puro sgFZD5 were generated after lentiviral infection and puromycin resistance selection. Single clones of Patu8988s, HPAF‐II, PANC‐1, and BxPC‐3 KO cells were generated by diluting the mixed KO cells at about 0.8 cell per well in 48‐well plates. The genotype of single‐cell clones was determined by amplifying the DNA fragments containing the sgRNA targeting region using the Fzd5‐RT‐qPCR primers (see Reagents and Resource) followed by ligating the PCR product into pCS2 vector (addgene). The ligation products were transformed into *E. coli* (NEB 5‐alpha strand) and plated onto agar plates. Multiple colonies were selected, and their plasmids were extracted and sequenced. The verified single clones were selected for further studies.

### Antibodies and Immunoblotting

The antibodies were purchased from various companies (see Reagents and Resource: Antibodies). The cell was lysed by Passive Lysis Buffer (25 mm Tris‐HCl, 150 mm NaCl, 1% NP40) containing a protease inhibitor cocktail (Roche). Total protein amount was determined by BCA assay (Thermo) and an appropriate amount of denatured protein with Lammeli loading dye was loaded onto 6% or 10% poly‐acrylamide gel for SDS‐PAGE. After that, PVDF membrane was used for transfer. And TBS‐T buffer containing 5% BSA with 0.02% sodium azide was used in membrane blocking and antibody incubations. For Western Blot, all primary antibodies were used in 1:1000 dilution and all secondary antibodies were used in 1:5000 dilution. The chemiluminescent substrate kit was purchased from GE and Tanon. Bound antibodies were visualized using a chemiluminescent substrate kit and exposed to Tanon 5200 and GE. Final quantification of gel intensity was done by ImageJ and plotted in Prism 8.0.

### Immunoprecipitation

For co‐immunoprecipitation, the total lysate of cell was subjected to *α*‐HA‐agarose, *α*‐Flag‐agarose, or *α*‐V5‐agarose at 4 °C overnight. Next day, the resins were washed thoroughly by TBS‐T for 5 times with 10 min incubation and shaking intervals at 4 °C before being subjected to SDS‐PAGE and Western Blot.

For Wnt3a and Fzd5 co‐immunoprecipitation, Wnt3a CM and the whole cell lysate containing Fzd5 were mixed and subjected to *α*‐V5‐agarose at 4 °C overnight. Next day, the resins were washed thoroughly by TBS‐T for 5 times with 10 min incubation and shaking intervals at 4 °C before being subjected to SDS‐PAGE and Western Blot.

### Immunofluorescence

HEK293T cells were grown on poly‐L‐lysine‐coated glass coverslips. Cells were transfected for 24 h and fixed in 4% paraformaldehyde (Sigma) for 30 min at RT. The fixed cells were washed twice with cold PBS, permeabilized, and blocked with 0.1% Triton X‐100/5% BSA/PBS for 30 min. The permeabilized cells were incubated overnight at 4 °C in the dark with primary antibody (1:200 dilution) and then washed by PBS 5 times, followed by incubation with secondary antibody at RT in the dark for 1 h (1:1000) and 5 times wash by PBS. Hoechst (Thermo) was next used to stain nuclei for 10 min, followed by 3 times washing by PBS. Samples were observed using inverted confocal microscopy (DM6000CS, Leica).

### Immunohistochemistry

Paraffin sections (5‐µm thickness) were deparaffinized and treated with 3% hydrogen peroxide for 10 min to quench the endogenous peroxidase activity. Antigenic retrieval was performed by submerging in citric acid (pH 6.0) and microwaving. The slides were then allowed to cool at RT, followed by incubation in normal goat serum for 1 h to block nonspecific binding, then incubated for 10 min with hematoxylin‐eosin staining or overnight at 4 °C using the following primary antibodies: *β*‐catenin (1:50, #8480, Cell Signaling Technology), Ki67 (1:400, #9449, Cell Signaling Technology). The staining was examined using HRP Envision Systems (DAB Kit, MXB Biotechnologies) and analyzed using a dissecting microscope (Leica DM4000).

### Reverse Transcription and Quantitative Real‐Time PCR

Total RNAs from cells were purified by UNlQ‐10 Column Trizol Total RNA Isolation Kit (Sango Biotech). cDNA was generated by using MonScript RTIII All‐in‐One Mix with dsDNase (Monad) according to the manufacturer's protocol. Quantitative RT‐PCR (qPCR, for RNA) and PCR (for genomic DNA) were performed using MonAmp ChemoHS qPCR Mix (Monad). All primers were designed based on the primer bank of Massachusetts General Hospital (https://pga.mgh.harvard.edu/cgi‐bin/primerbank). The reaction mixtures were incubated at 50 °C for 15 min, followed by 95 °C for 5 min, and then 35 PCR cycles were performed with the following temperature profiles: 95 °C for 15 s, 60 °C for 30 s, and 72 °C for 1 min. The gene expression values were normalized to those of GAPDH. PCR primer sequences were listed in the “Reagents and Resource”. The statistical analysis was done by One‐way ANOVA in Prism 8.0.

### TOP‐FLASH Dual‐Luciferase Reporter Assay

For Top‐Flash reporter assay, Top‐Flash reporter cell line (HEK293T B/R, cells harbor stably expressing superTOP‐Flash (BAR) and Renilla (internal control) vectors) were used.

Cells were plated in 24 well plates and transfected the following day in triplicate using Lipofectamine 2000. Dual‐luciferase reporter assays were performed using Dual‐Luciferase Reporter Gene Assay Kit (Beyotime Biotechnology) according to manufacturer's instructions. The test‐ready plate was assayed by a Biotek Synergy H1 microplate reader. Representative results were shown from three (or more) independent experiments. The statistical analysis was done by One‐way ANOVA (for three or more groups) or two‐tailed unpaired student's t‐test (for two groups) in Prism 8.0.

### Pulse‐Chase Assay

The cells in 24‐well plates (with 70–80% confluency) were treated with cycloheximide (CHX) 300 µg mL^−1^ in a series of time points (i.e., 0, 2, 4, 6, 8, and 10 h). After the treatment, the cells were washed, harvested, and lysed to extract proteins, and the cell lysate was subjected to WB as described above. Afterwards, the gel intensity was quantified by ImageJ and fitted into first‐order decay, and protein half‐life (t_1/2_) was calculated according to ln(N)−ln(N_0_) = −kt linear equation. k = degradation rate constant (min^−1^). The statistical analysis was done by a two‐tailed unpaired student's t‐test in Prism 8.0.

### 22‐NHC Conjugated and Fatty Acid Conjugated Beads Synthesis

The 22‐NHC conjugated agarose beads and the control beads were produced as previously described.^[^ ^10a^ ^]^

### Mass‐Spectrometry and FT‐IR Assay

The His6‐V5‐tagged Fzd5 and Fzd7 were constructed and transfected into HEK293T in 10‐cm petri dishes. The cells were harvested after 48‐h transfection and lysed with the buffer containing 20 mm HEPES, 150 mm NaCl, and 0.025% digitonin, pH 7.5. Afterwards, the cell lysate was subjected to Ni‐NTA beads for enrichment, and washed thoroughly by the abovementioned buffer for 5 times. After washing, the buffer was removed and 3 m urea was added to the beads to denature the proteins. The mixture was then transferred to a glass vial where dichloromethane was added to extract the hydrophobic substance. After 4 h extraction by gentle shaking, the organic layer was separated by a glass pipette and dried with nitrogen gas.

For Mass‐spectrometry assay, the samples were loaded with the solvent containing 90% methanol, 10% water, and 0.1% formic acid. The samples were subjected to Agilent Q‐TOF 6540 with Dual AJS ESI ion mode and 0.200 mL min^−1^ flow rate. 50–1000 (m/z) was acquired with a scan rate of 3.00 spectra s^−1^. For FT‐IR assay, the samples were dissolved in methanol and subjected to Bruker Tensor 27 FT‐IR spectrometer. The data of Mass‐spectrometry and FT‐IR were plotted in Origin 2019 after acquisition.

### Conjugated Beads Pull‐Down and Competition Assay

The cell lysate containing the desired protein was clarified by centrifugation at 13 000 rpm for 30 min at 4 °C. The supernatant was then first incubated with the desired competitor compound or ethanol control for 15 min on ice. All of the compounds were added to binding reactions from ethanol stock solutions. After this incubation, 22‐NHC beads or control beads were added, followed by end‐over‐end rotation for 1 h at 4 °C. The beads were washed three times with wash buffer (20 mm HEPES, pH 7.5, 150 mm NaCl, 0.1% NP40). Afterwards, the bound proteins were eluted in SDS‐PAGE sample buffer with DTT (50 mm final) at 37 °C. The proteins were then separated by SDS‐PAGE, followed by Western blot. A portion of the clarified cell lysate was used as input control in Western blot.

### Methyl Thiazolyl Tetrazolium (MTT) Assay for Cell Proliferation

Cells were plated on 96‐well plates at a density of 1000 cells per well in triplicates and incubated for 1–10 days. MTT (thiazolyl blue tetrazolium, from Sigma) was added to each well for a final concentration of 0.5 mg mL^−1^ and the plate were incubated at 37 °C for an additional 4 h. After the incubation, all medium was removed and 100 µL of DMSO was added to each well. Then the test‐ready plate was assayed by Biotek Synergy H1 microplate reader at OD_490_. Growth curve was drawn according to OD_490_ values by days. The statistical analysis was done by Two‐way ANOVA in Prism 8.0.

### Colony Formation Assay

Patu8988s and HPAF‐II cells seeded in 6‐well plates at 2000 cells/well on the third day after infection were cultured for 14 days to form colonies. Cells were subsequently treated by washing with PBS, fixing in 4% paraformaldehyde for 15 min, staining with 0.5% crystal violet for 1 h, washing three times by ddH_2_O, and photographing with a digital camera. All assays were performed in triplicate.

### Growth of Cells in Athymic Nude Mouse and Tumor Size Determination

Patu8988s cells were trypsinized, washed, and resuspended in PBS. Designed cell number and viability were determined using trypan blue. Thirty 6‐week old female athymic nude mice (Beijing HFK Bioscience CO., LTD) were randomly divided into three groups (10 mice/group) followed by subcutaneously injecting with Patu8988s cells at a density of 1× 10^6^ cells per site. The tumor size was determined every 2–3 days. Tumor volumes were measured, and the mice were weighed twice weekly. Tumor volume was calculated using the formula: ½ (Length × Width^2^). The tumor weight was measured after sacrificing the nude mice and dissecting the tumor. The statistical analysis for tumor volume was done by Two‐way ANOVA and the statistical analysis for tumor weight was done by One‐way ANOVA in Prism 8.0.

### Tumor Tissues Protein Extraction

The tumor tissues in RIPA buffer (Cowin Bio) were disrupted using an ultrasonic disruptor (SCIENTZ) until no obvious tissue mass was present. The disrupted tumor tissues were lysed in RIPA buffer for 2 h at 4 °C. And then the tissue lysate was clarified by centrifugation at 13 000 rpm for 25 min at 4 °C. Total protein amount was determined by BCA assay (Thermo) and appropriate amounts of protein were used in further assays.

### Cholesterol Starvation, Depletion, and Rescue Assay

Cholesterol starvation was performed on cells with 70–80% confluency. Pravastatin (20 µm) (Sigma) was incubated with cells cultured in Lipoprotein Deficient Serum (Sigma) for 24 h before lysis. Cholesterol depletion was performed on starved, confluent cells. The cells were incubated for 30 min with 1% methyl‐*β*‐cyclodextrin (M*β*CD) in DMEM, after which subsequent incubations were done in DMEM with 20 µm pravastatin. For cholesterol rescue experiments, cholesterol was added back by incubating the cells for 1 h with soluble cholesterol‐M*β*CD complexes (100 µm in DMEM supplemented with 40 µm pravastatin) after either cholesterol starvation or depletion. When the assays were finished, the cells were lysed and subjected to immunoblotting. For cholesterol supplementation in cell growth both 2D or 3D, cholesterol‐M*β*CD complexes were added at the final concentration of 10ug mL^−1^ in the culture medium.

### Surface Biotin Labeling Assay

Cell surface proteins were biotinylated using Sulfo‐NHS‐SS‐Biotin (APExBIO) according to the manufacturer's protocol. Biotinylated proteins were enriched by Pierce Streptavidin Agarose.

### SNAP‐Tag Labeling and Fluorescent Assay

For SNAP‐tag labeling, HEK293T cells were grown on poly‐L‐lysine‐coated glass coverslips. Cells were transfected for 24 h and then labeled with SNAP‐Surface 549 (NEB) or SNAP‐Cell Oregon Green (NEB) for 30 min at 37 °C according to the manufacturer's protocol. Subsequently, cells were washed three times with cold PBS and fixed in 4% paraformaldehyde (Sigma). Hoechst (Thermo) was used to stain nuclei. Finally, the samples were visualized using a confocal microscope (DM6000CS, Leica).

### Cellular Content Fractionation

Using digitonin lysis buffer (25 mm Tris‐HCl, 150 mm NaCl, 0.015% digitonin) to separate cell membrane and cytoplasm: first, cells were lysed by using digitonin lysis buffer for 10 min and centrifuged at 8000 rpm for 2 min at 4 °C. The supernatant was collected first (cytoplasmic fraction). The pellet was then lysed by Passive Lysis Buffer.

Using Minute Plasma Membrane Protein Isolation and Cell Fractionation Kit (Inventbiotech) to separate different cell fractions: according to the manufacturer's protocol.

### Acyl‐PEG Exchange Assay

The Acyl‐PEG exchange assay (APE) was described.^[^ ^20^ ^]^ In brief, cell samples were lysed and the protein concentration of the cell lysate was then measured using a BCA assay (Thermo) and adjusted to 2 mg mL^−1^ with lysis buffer. Typically, 200 µg of total protein in 92.5 µL of lysis buffer was treated with 5 µL of 200 mm neutralized TCEP (Sigma) for a final concentration of 10 mm TCEP for 30 min with nutation. NEM (Sigma), 2.5 µL from freshly made 1 m stock in ethanol, was added for a final concentration of 25 mm and incubated for 2 h at RT. Reductive alkylation of the proteins was then terminated by methanol‐chloroform‐H_2_O precipitation (4:1.5:3). For hydroxylamine (NH_2_OH) cleavage and mPEG‐maleimide alkylation, the protein pellet was resuspended in 30 µL TEA buffer containing 4% SDS, 4 mm EDTA and treated with 90 µL of 1 m neutralized NH_2_OH (Sigma) dissolved in TEA buffer pH 7.3, containing 0.2% Triton X‐100 (Sigma) to obtain a final concentration of 0.75 m NH_2_OH. Control samples not treated with NH_2_OH were diluted in 90 µL TEA buffer with 0.2% Triton X‐100. Samples were incubated at RT for 1 h with nutation. The samples were then subjected to methanol‐chloroform‐H_2_O precipitation and resuspended in 30 µL TEA buffer containing 4% SDS, 4 mm EDTA, warmed to 37 °C for 10 min, and briefly (≈5 s) sonicated and treated with 90 µL TEA buffer with 0.2% Triton X‐100 and 1.33 mm mPEG‐Mal (10 kDa; Sigma) for a final concentration of 1 mm mPEG‐Mal. Samples were incubated for 2 h at RT with nutation before a final methanol‐chloroform‐H_2_O precipitation. Dried protein pellets were resuspended in 50 µL lysis buffer and then heated for 5 min at 95 °C. Typically, 15 µL of the sample was analyzed by Western Blot.

### Click Chemistry and Pull‐Down Assay

Cells were treated with 50 µm palmitic acid analog Alk‐16 (Sigma) for 5 h and collected and lysed in 1% NP‐40 lysis buffer (25 mm Tris‐HCl pH 8.0, 150 mm NaCl, 10% glycerol, 1% NP‐40) with protease inhibitor cocktail (Roche). The supernatant was collected after centrifugation at 14 000 rpm for 20 min at 4 °C. The protein concentration was determined by BCA assay (Thermo). Click chemistry reagents were added to the beads in the following order: 1 µL of 10 mm Biotin‐azide (APExBIO), 1.2 µL of 10 mm tris[(1‐benzyl‐1H‐1,2,3‐triazol‐4‐yl)methyl]amine (TBTA) (Sigma), 1 µL of 40 mm CuSO_4_, and 1 µL of 40 mm tris(2‐carboxyethyl)phosphine HCl (TCEP hydrochloride) (Sigma). The reaction mixtures were mixed thoroughly and incubated for 30 min in the dark at RT. 10 µL of 0.5 m EDTA was added to quench the reaction and the sample was briefly vortexed. The pellet was resuspended in 50 µL TEA buffer and vortexed for 10 min at RT. Once the pellet was resuspended, 450 µL TEA buffer was added and vortexed briefly. 15 µL of Pierce Streptavidin Agarose (Pierce) was washed three times with TEA buffer and added to the supernatant from each sample and the mixture was incubated at RT with end‐over‐end rotation for 90 min. The beads were washed with 1 mL 1% SDS in PBS three times. Each sample was resuspended in 50 µL lysis buffer and then heated for 5 min at 95 °C. Typically, 15 µL of the sample was analyzed by Western Blot.

### Cellular Cholesterol Extraction and Quantification

Cellular cholesterol content was extracted with chloroform, methanol, and 0.9% NaCl as described previously.^[^ ^14a^ ^]^ Briefly, for thin‐layer chromatography (TLC), 5 µL of the sample were spotted on silica gel TLC plates. The plate was developed in petroleum‐ether ethyl‐acetate (1:2). After the plate was sprayed with 1% anisaldehyde‐sulfuric acid, cholesterol content was visualized on Tanon 5200. Final quantification of cholesterol content intensity was done by ImageJ. The statistical analysis was done by a two‐tailed unpaired student's t‐test in Prism 8.0.

### L Conditioned Medium (LCM) and L‐Wnt3a Conditioned Medium (Wnt3aCM) Preparation

L cells and L‐Wnt3a cells were grown in DMEM Medium supplemented with 10% FBS. The conditioned medium was collected after the cell confluency reached 80–90% for 3 days.

### Glycosylation Site Prediction

Glycosylation site prediction was performed by the online tool website below:http://www.cbs.dtu.dk/services/NetNGlyc/

### RNAseq Analysis

The RNAseq data of TCGA‐PAAD was downloaded from Broad GDAC Firehose platform. For the 177 cases (primary tumor) with RNA‐Seq gene expression data, gene expression files with RSEM expression values associated with 20154 gene symbols were available. These values were combined into a single file.

Based on the information on the cBioPortal, 11 cases were classified into the RNF43‐mutant group and the rest of the 166 primary tumor cases as RNF43‐WT group. 1294 DEGs in RNF43‐mutant group (145 up‐regulated and 1149 down‐regulated genes) were identified in RStudio with DESeq2 package, with *p*‐value < 0.05 and fold change (FC) ≥ 1 and base mean ≥ 5. Gene Ontology (GO) and KEGG pathway enrichment analysis for 1294 DEGs were performed by R package cluster profiler with default settings (*p*‐value < 0.05).

### Primary Tumor Sample DNA Extractions, Targeted Sequencing, and Data Processing

DNA extraction and sequencing were performed as previously described with some modifications.^[^ ^27^ ^]^ QIAamp DNA Micro Kit (Cat No./ID: 56304) was used to extract genomic DNA from tissue samples, and NanoDrop2000 was used to detect the concentration and purity of DNA. DNA was segmented to a length range of 150–300 bp using Covaris M220 DNA ultrasonic fragmentation instrument. The fragment DNA was amplified after terminal repair and the addition of A reaction. XGen Exome Research Panel V1.0 kit (IDT) was used to target the amplified library. PE150 sequencing was performed using Illumina Novaseq 6000. Bioinformatics analysis: BWA was used to compare reads to the human reference genome (GRCH37/HG19),^[^ ^28^ ^]^ and GATK standard mutation detection process was used to detect SNP and INDEL.^[^ ^29^ ^]^ The results of the variation obtained were annotated by ANNOVAR.^[^ ^30^ ^]^ In the screening process of mutation loci, mutations with median gene frequencies greater than 1% in gnomAD, ExAC, 1000 database, and ESP6500 database were filtered out. It then prioritizes them in order to study potentially harmful variants. Mutation loci were classified according to ACMG genetic variation classification criteria and guidelines.^[^ ^31^ ^]^ Genomic profiling was performed in a Clinical Laboratory Improvement Amendments‐certified and College of American Pathologists‐accredited laboratory (3D Medicines, Inc., Shanghai, China; Aegicare (Shenzhen) Technology Co., Ltd., Shenzhen, China; GeneCast Biotechnology Co. Ltd., Wuxi, China)

### Untargeted Liposome Analysis of PDAC Tumor Tissue

Cancer tissues were collected from 12 patients with PDAC. The samples were put into liquid nitrogen for liposome analysis. Lipid metabolites were extracted from 25 mg tissue samples using the extract solution (MTBE: MeOH = 5:1) for non‐targeted LC‐MS/MS analysis, and the data were processed and annotated. The UHPLC separation was carried out using a 1290 Infinity series UHPLC System (Agilent Technologies), equipped with a Kinetex C18 column (2.1 * 100 mm, 1.7 µm, Phenomen). The Triple TOF mass spectrometer was used for its ability to acquire MS/MS spectra on an information‐dependent basis (IDA) during an LC/MS experiment. An in‐house program, namely, LipidAnalyzer, was developed using R for automatic data analysis. The raw data files (.wiff format) were converted to files in mzXML format using the “msconvert” program from ProteoWizard (version 3.0.6150). Then, the mzxML files were loaded into LipidAnalyzer for data processing. Peak detection was first applied to the MS1 data. The CentWave algorithm in XCMS was used for peak detection, With the MS/MS spectrum, lipid identification was achieved through a spectral match using an in‐house MS/MS spectral library. The detection and analysis of liposomes were carried out by BIOTREE, Shanghai, China.^[^ ^32^ ^]^ Quantification results were shown as the mean ± SEM, two‐tailed unpaired student's t‐test was performed for statistical analysis in Prism 8.0.

### Heatmap Generation

The raw Mass‐spec data from different patients were normalized (set scale = “row”) and the heatmap was generated by R package (pheatmap). Values represent the result of centralizing (subtracting the row means from their corresponding rows) and scaling (dividing the centralized rows by their standard deviations) the original data.

### Image Quantification

Image quantification was performed by Image J: Using Image J→ Analyze→Gels→Plot Lanes to analyze the Western Blot results.

### Pulse‐Chase Assay

The values were calculated according to the image quantification, the 0 h value was used as the control, and the values at all time‐points were divided by the 0 h value to obtain the results for image drawing. Two‐tailed unpaired student's t‐test was performed for statistical analysis.

### TOP‐FLASH Assay

After the values were obtained according to the TOP‐FLASH Dual‐luciferase reporter assay method, the fluorescence values of Firefly were divided by the corresponding fluorescence values of Renilla (internal control). The values of EV + LCM were used as control, and all the values were divided by the values of EV + LCM for normalization, and the obtained results were used for image drawing. Experimental results were shown as the mean ± SD, n = 3 replicates, one‐way ANOVA (for three or more groups), or two‐tailed unpaired student's t‐test (for two groups) was performed for statistical analysis.

### MTT Assay

The OD_490_ values at each time point were used as the result for image drawing. Experimental results were shown as the mean ± SD; n = 3 replicates; two‐way ANOVA was performed for statistical analysis.

### Quantitative Real‐Time PCR

The gene expression values were normalized to those of GAPDH. And data processing was performed using the 2^−ΔΔCt^ method, the results were calculated for image drawing. Experimental results were shown as the mean ± SD; n = 3 replicates; one‐way ANOVA was performed for statistical analysis.

### Tumor Volumes

Tumor volume was calculated using the formula: ½ (Length × Width^2^). Images were drawn after obtaining the tumor volumes in combination with the corresponding time points. Experimental results were shown as the mean ± SD; n = 10 for each group; two‐way ANOVA was performed for statistical analysis.

### Tumor Weight

The tumor weight was measured after sacrificing the nude mice and dissecting the tumor. Images were drawn based on the weight results. Experimental results were shown as the mean ± SD; n = 10 for each group; one‐way ANOVA was performed for statistical analysis.

### S‐Palmitoylated Protein in Tumors

The blotting intensity was quantified by ImageJ as described above. The palmitoylated band intensity was then divided by the total band intensity (palmitoylated plus unpalmitoylated bands) to calculate the percentage of palmitoylated proteins. Experimental results were shown as the mean ± SD; n = 10 for high cholesterol diet and normal diet group, n = 9 for statin‐treated group; one‐way ANOVA was performed for statistical analysis.

### Cellular Cholesterol Quantification in PDAC Cell Line

Image quantification was performed by Image J. The standard sample was used as the control, and all the values were divided by the values of the standard sample for normalization, and the obtained results were used for image drawing. Experimental results were shown as the mean ± SD; n = 3 replicates; two‐tailed unpaired student's t‐test was performed for statistical analysis.

### Cholesterol Metabolites Quantification in PDAC Primary Tumors

Contents of cholesterol metabolites were plotted using GraphPad Prism 8.0 for scatter plots. Experimental results were shown as the mean ± SEM; two‐tailed unpaired student's t‐test was performed for statistical analysis.

*p*‐values < 0.05 were considered to be statistically significant. GraphPad Prism 8.0 software was used for statistical analyses.

## Conflict of Interest

The authors declare no conflict of interest.

## Author Contributions

R.S. and M.W. designed the study. S.Z., J.L., Z.P., H.Z., Y.W., L.M., Ha.Z., M.C., J.Q., C.Z. M.W., and R.S. performed experiments and collected and analyzed the data. S.Z., M.C., X.Z., L.C., Xin.Z., M.W., X.H., and R.S. wrote and revised the manuscript. L.C., M.W., X.H., and R.S. oversaw the study. All authors have approved the manuscript.

## Supporting information

Supporting Information

Supporting Table 1

Supporting Table 2

## Data Availability

The data that support the findings of this study are available from the corresponding author upon reasonable request.
