# Supplementary material for: Aberrant Cholesterol Metabolism and Wnt/β‐Catenin Signaling Coalesce via Frizzled5 in Supporting Cancer Growth
Source: Adv Sci (Weinh). 2022 Aug 17;9(28):2200750. doi: 10.1002/advs.202200750 (PMC9534957; doi:10.1002/advs.202200750)
Supplement: Supplementary file 1 — Supporting Information [file ADVS-9-2200750-s003.pdf]

Supporting Information

Aberrant cholesterol metabolism and Wnt/β-catenin signaling coalesce via Frizzled5 in supporting cancer growth

Shaoqin Zheng, Jiahui Lin, Zhongqiu Pang, Hui Zhang, Yinuo Wang, Lanjing Ma, Haijiao Zhang, Xi Zhang, Maorong Chen, Xinjun Zhang, Chao Zhao, Jun Qi, Liu Cao, Min Wang, Xi He*, Ren Sheng*.*

Supplemental figures and legends

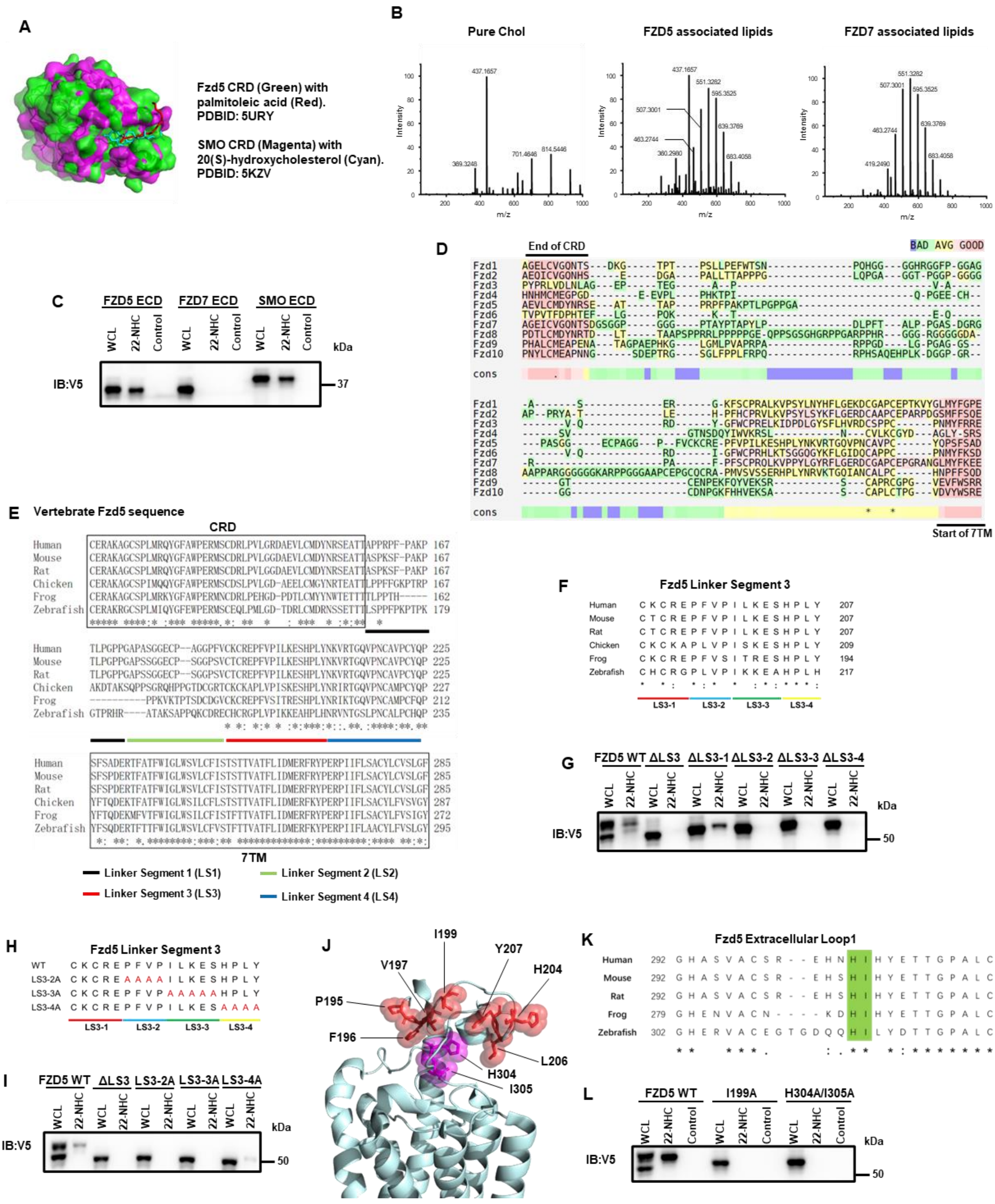

Supplemental Figure 1. Supporting information and assays of Fzd5-cholesterol bindings. A) Overlay of Fzd5 and Smo CRDs by Pymol (PDBID: 5URY and 5KZV). B) Full spectra (m/z range: 50-1000) of mass-spectrometry assay of pure cholesterol, Fzd5-associated lipids and Fzd7-associated lipids. C) 22-NHC pulldown and WB assay of secreted Fzd5, Fzd7 and Smo ECDs. D) Amino acid sequence alignment of human Fzd1-10 extracellular linker region by T-coffee (http://tcoffee.crg.cat/). The different conservation levels are highlighted by different colors. The result shows that the extracellular linker region is not conserved among Fzds. E) Amino acid sequence alignment by clustalW of the extracellular linker region of vertebrate Fzd5 and demonstration of LS1, LS2, LS3 and LS4. F) Amino acid sequence alignment of Fzd5 LS3 in vertebrate and demonstration of LS3-1, LS3-2, LS3-3 and LS3-4. G) 22-NHC beads pulldown assay of Fzd5 WT, ΔLS3 and smaller truncations (ΔLS3-1, ΔLS 3-2, ΔLS 3-3 and ΔLS 3-4). H) Demonstration of LS3-2A, LS3-3A and LS3-4A (Replacing all amino acids with Ala). I) 22-NHC beads pulldown and WB assay of Fzd5 WT and mutations in LS3. J) Demonstration of conserved residues on LS3 that involves in cholesterol binding (red) and adjacent aromatic/hydrophobic conserved residues in ECL1 (pink) based on the Fzd5 structure model (PDBID: 6WW2). K) Sequence alignment of Fzd5 ECL1 among vertebrates. The conserved His and Ile are highlighted in green. L) 22-NHC beads pulldown assay of Fzd5 WT, I199A and H304A/I305A.

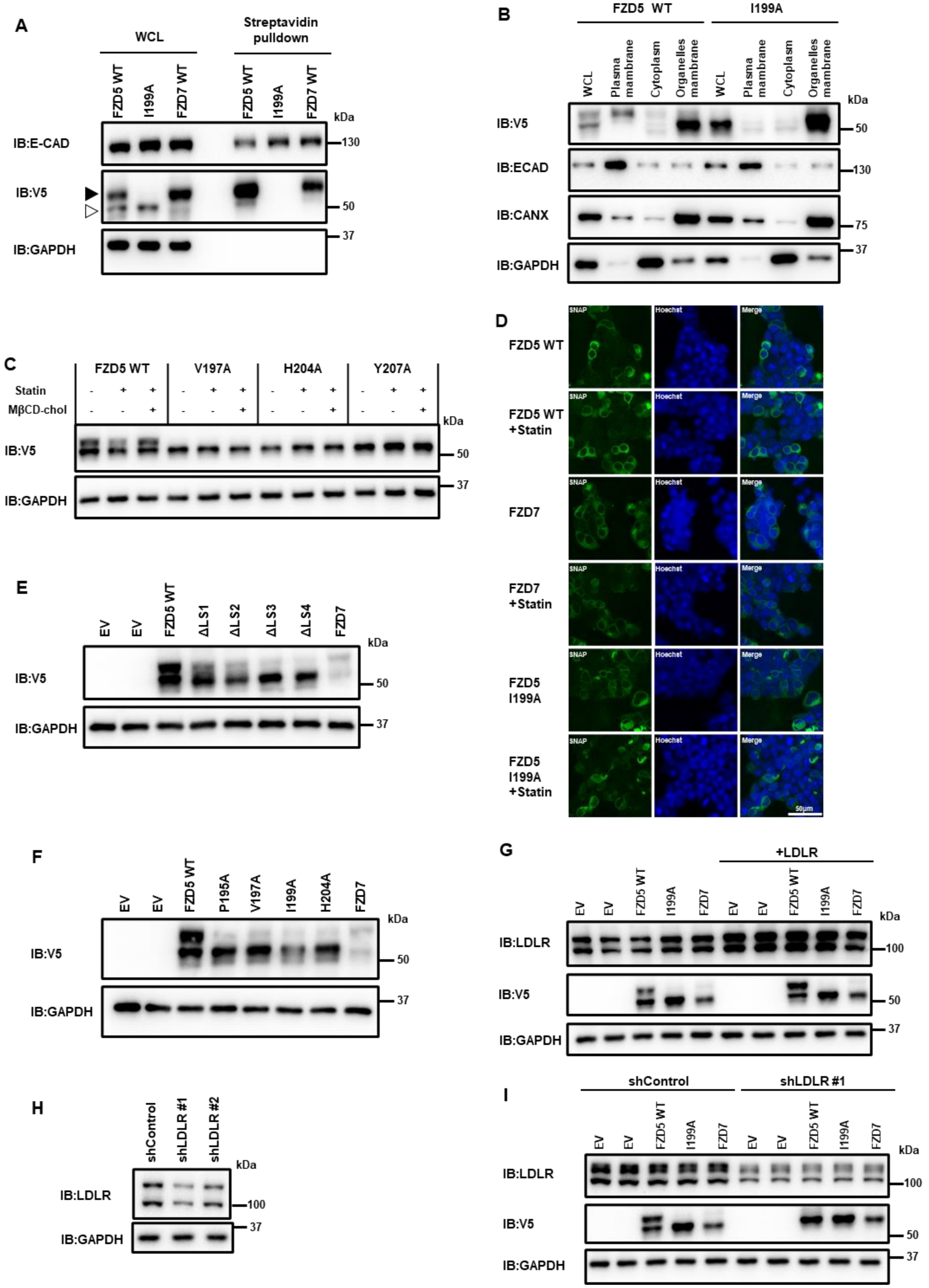
A
WCL
Streptavidin pulldown
FZD5 WT
I199A
FZD7 WT
kDa
IB:E-CAD
IB:V5
IB:GAPDH
130
50
37
B
FZD5 WT
I199A
WCL
Plasma mambrane
Cytoplasm
Organelles mambrane
IB:V5
IB:ECAD
IB:CANX
IB:GAPDH
75
C
V197A
H204A
Y207A
Statin
MβCD-chol
D
SNAP
Hoechst
Merge
FZD5 WT
FZD5 WT +Statin
FZD7
FZD7 +Statin
FZD5 I199A
FZD5 I199A +Statin
50μm
E
EV
ΔLS1
ΔLS2
ΔLS3
ΔLS4
FZD7
F
P195A
G
+LDLR
IB:LDLR
100
H
shControl
shLDLR #1
shLDLR #2
I

Supplemental Figure 2. Cholesterol promotes Fzd5 PM localization and Wnt signaling. A) Surface biotin labeling assay of Fzd5 WT, I199A and Fzd7. B) Cellular fractionation assay of Fzd5 WT and I199A in HEK293T cells. E-CAD: PM marker. CANX: inner membrane marker. GAPDH: cytoplasm marker. C) Mature/immature band pattern of Fzd5 WT and other cholesterol binding LoF mutations besides I199A in HEK293T cells. D) Confocal fluorescent imaging of total SNAP-tagged Fzd5, Fzd7 and I199A under normal and statin treatment conditions in HEK293T cells. SNAP-tagged proteins are labelled with cell-permeable SNAP Cell Oregon Green. Hoechst labels the nucleus. All images in the panel are in the same scale. E) Protein expression of the TOPFlash assay in Figure 2D in Fzd-null HEK293T. F) Protein expression of the TOPFlash assay in Figure 2E in Fzd-null HEK293T. G) Protein expression of the TOPFlash assay in Figure 2K in Fzd-null HEK293T. H) Validation of LDLR knockdown by WB in Fzd-null HEK293T. I) Protein expression of the TOPFlash assay in Figure 2L in Fzd-null HEK293T.

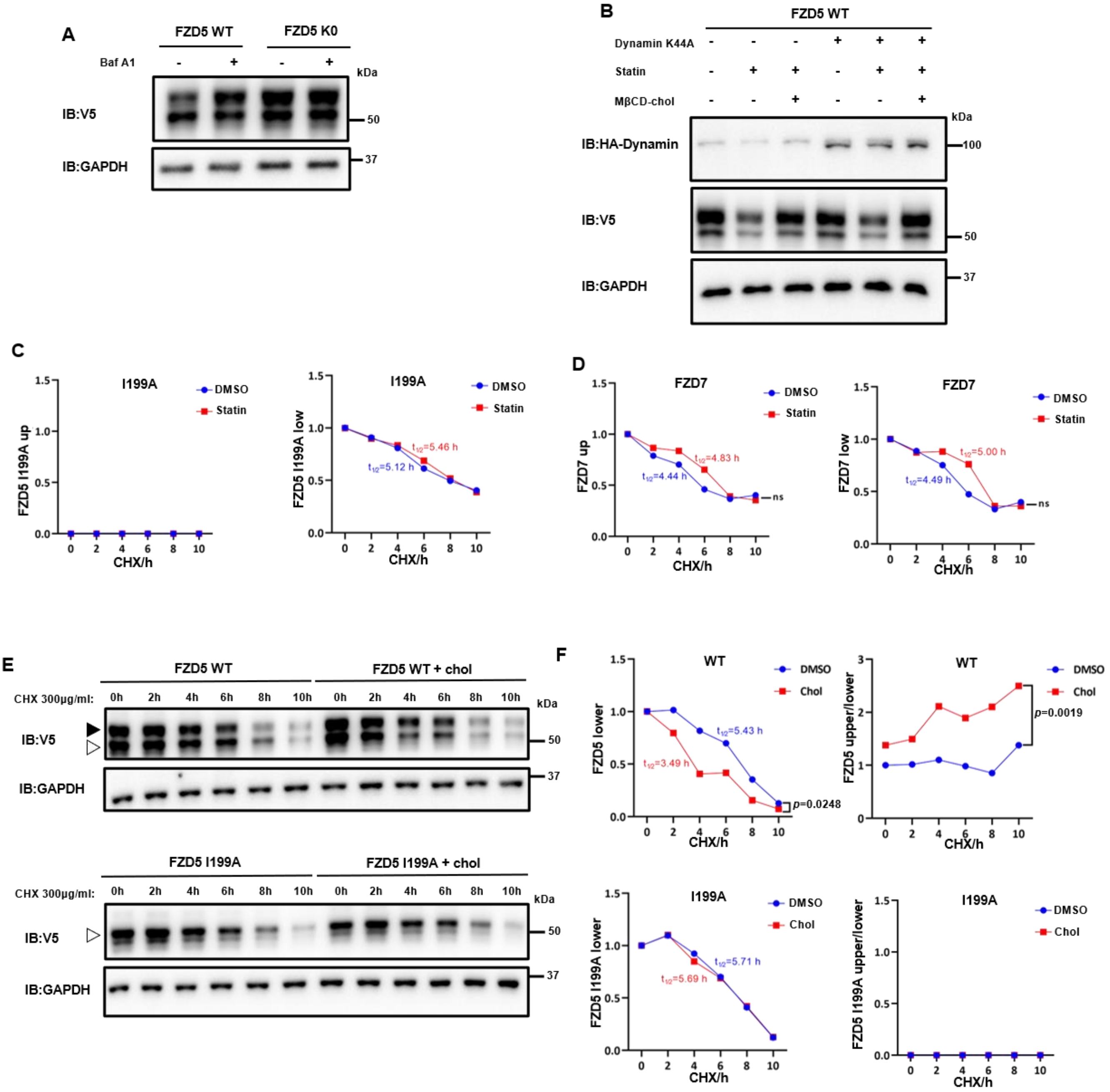
A
FZD5 WT
FZD5 K0
Baf A1
kDa
IB:V5
50
IB:GAPDH
37
B
FZD5 WT
Dynamin K44A
Statin
MβCD-chol
kDa
IB:HA-Dynamin
100
IB:V5
50
IB:GAPDH
37
C
I199A
DMSO
Statin
FZD5 I199A up
CHX/h
I199A
FZD5 I199A low
t1/2=5.46 h
t1/2=5.12 h
D
FZD7
FZD7 up
t1/2=4.83 h
t1/2=4.44 h
ns
FZD7
FZD7 low
t1/2=5.00 h
t1/2=4.49 h
ns
E
FZD5 WT
FZD5 WT + chol
CHX 300μg/ml:
0h 2h 4h 6h 8h 10h
IB:V5
IB:GAPDH
FZD5 I199A
FZD5 I199A + chol
F
WT
Chol
FZD5 lower
t1/2=5.43 h
t1/2=3.49 h
p=0.0248
WT
FZD5 upper/lower
p=0.0019
I199A
FZD5 I199A lower
t1/2=5.71 h
t1/2=5.69 h
I199A
FZD5 I199A upper/lower

Supplemental Figure 3. Supporting assays of cholesterol regulating Fzd5 independent of ubiquitination-lysosomal degradation. A) Treatment of Baf A1 on Fzd5 WT and K0 in HEK293T. B) WB assay showing cholesterol does not affect endocytosis by employing dominant negative dynamin in HEK293T. C) The quantification by ImageJ of the upper/lower protein and the half-lives in Figure 3H. D) The quantification by ImageJ of the upper/lower protein and the half-lives in Figure 3I. By two-tailed unpaired student's t-test analysis. E) Pulse chase assay of Fzd5 WT and I199A under normal and additional cholesterol treatment conditions. The solid arrow head represents the upper mature Fzd and the hollowed arrow head represents the lower immature Fzd. F) The quantification by ImageJ of the protein and upper/lower ratio of the blots in E). By two-tailed unpaired student's t-test analysis. ns: not significant.

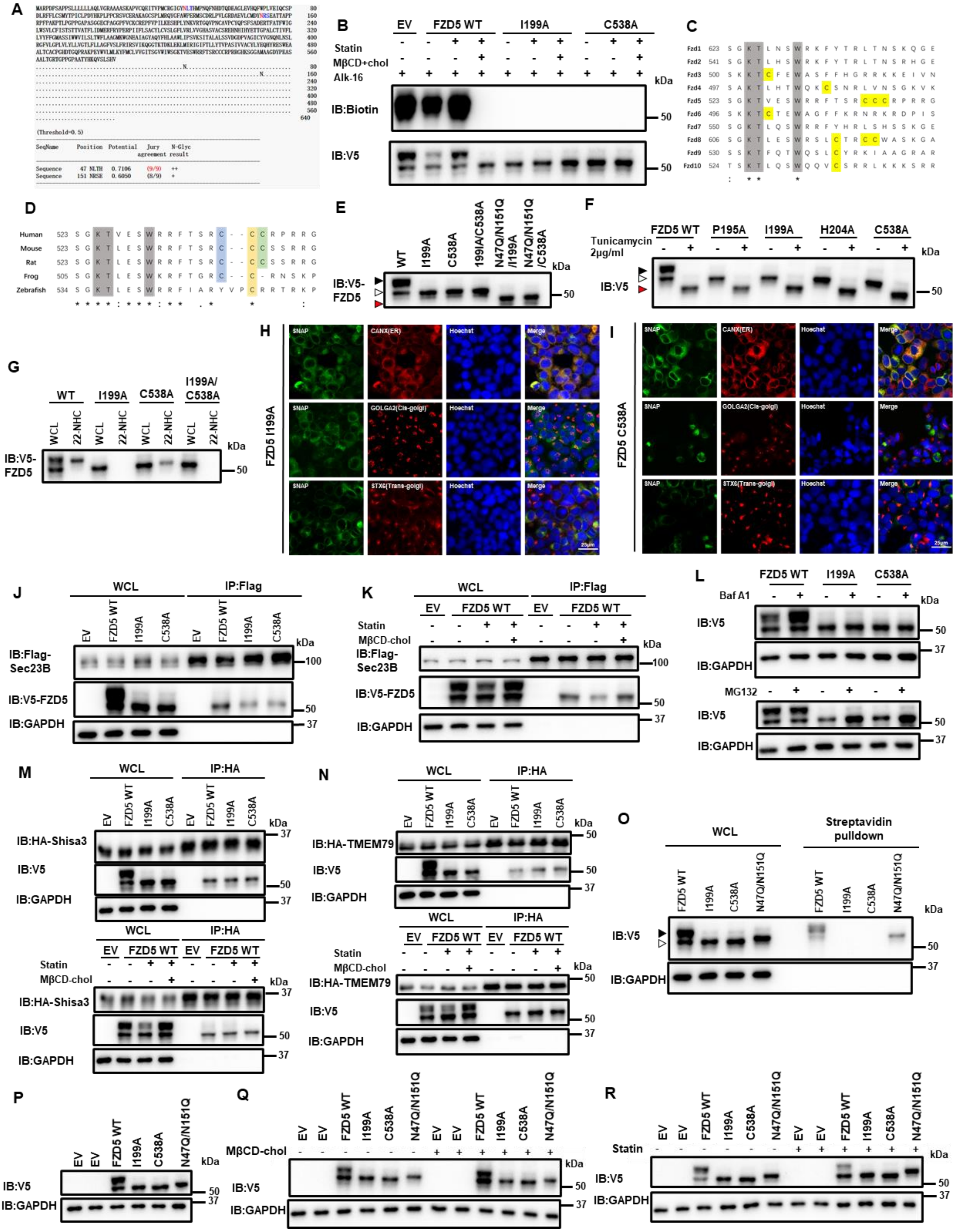
A
B
EV
FZD5 WT
I199A
C538A
Statin
MβCD+chol
Alk-16
kDa
IB:Biotin
IB:V5
50
C
Fzd1
Fzd2
Fzd3
Fzd4
Fzd5
Fzd6
Fzd7
Fzd8
Fzd9
Fzd10
D
Human
Mouse
Rat
Frog
Zebrafish
E
WT
I199A
C538A
I199A/C538A
N47Q/N151Q /I199A
N47Q/N151Q /C538A
IB:V5-FZD5
F
Tunicamycin 2μg/ml
P195A
H204A
G
I199A/ C538A
WCL
22-NHC
H
FZD5 I199A
SNAP
CANX(ER)
Hoechst
Merge
GOLGA2(Cis-golgi)
STX6(Trans-golgi)
I
FZD5 C538A
J
IP:Flag
IB:Flag-Sec23B
IB:V5-FZD5
IB:GAPDH
100
37
K
MβCD-chol
L
Baf A1
MG132
M
IP:HA
IB:HA-Shisa3
N
IB:HA-TMEM79
O
Streptavidin pulldown
N47Q/N151Q
P
Q
R

Supplemental Figure 4. Supporting analysis and assays of the cholesterol binding dependent S-palmitoylation of Fzd5, maturation and Wnt signaling. A) N-glycosylation prediction of Fzd5. B) Click chemistry and pulldown assay showing Fzd5 palmitoylation by Alkyne-palmitic acid. C) Sequence alignment of the cytoplasmic tail of 10 Fzd receptors. KTxxxW motif is highlighted in grey. Cysteines are highlighted in yellow. The three-in-a-row Cys pattern is unique for Fzd5. D) Sequence alignment of the cytoplasmic tail of Fzd5 among vertebrates. KTxxxW motif is highlighted in grey. Three-in-a-row Cys pattern is conserved among mammals and at least one Cys at the cytoplasmic tail is conserved in vertebrates. E) Band pattern of Fzd5 WT and mutants. The solid arrow head represents the upper mature Fzd. The hollowed arrow head represents the lower immature Fzd. The red solid arrow head represents the naïve Fzd without modifications. F) Band pattern of Fzd5 WT and mutants under the treatment of tunicamycin. The solid arrow head represents the upper mature Fzd. The hollowed arrow head represents the lower immature Fzd. The red solid arrow head represents the naïve Fzd without modifications. G) 22-NHC beads pulldown assay of Fzd5 WT, I199A, C538A, I199A/C538A. H, I) Confocal fluorescent microscopic images showing Fzd5 WT subcellular localizations of Fzd5 I199A H) and Fzd5 C538A I) in HEK293T. SNAP-Fzd5 is labelled by cell-permeable dye. CANX, GOLGA2 and STX6 staining are immunofluorescent. All images are in the same scale. J) Co-immunoprecipitation of Fzd5 WT, I199A and C538A with COPII complex subunit Sec23B. K) Co-immunoprecipitation of Fzd5 under normal, statin treatment and cholesterol rescue conditions with Sec23B. L) Fzd5 WT, I199A and C538A stability test under Baf A1 and MG132 treatment. M) Co-immunoprecipitation assay of Fzd5 WT and mutants with Shisa (upper) or Fzd5 WT with Shisa during cholesterol perturbations (lower). N) Co-immunoprecipitation assay of Fzd5 WT and mutants with TMEM79 (upper) or Fzd5 WT with TMEM79 during cholesterol perturbations (lower). O) Surface biotin labeling of Fzd5 WT, I199A, C538A and N47Q/N151Q. P) Protein expression of the TOPFlash assay in Figure 4J in Fzd-null HEK293T. Q) Protein expression of the TOPFlash assay in Figure 4K in Fzd-null HEK293T. R) Protein expression of the TOPFlash assay in Figure 4L in Fzd-null HEK293T.

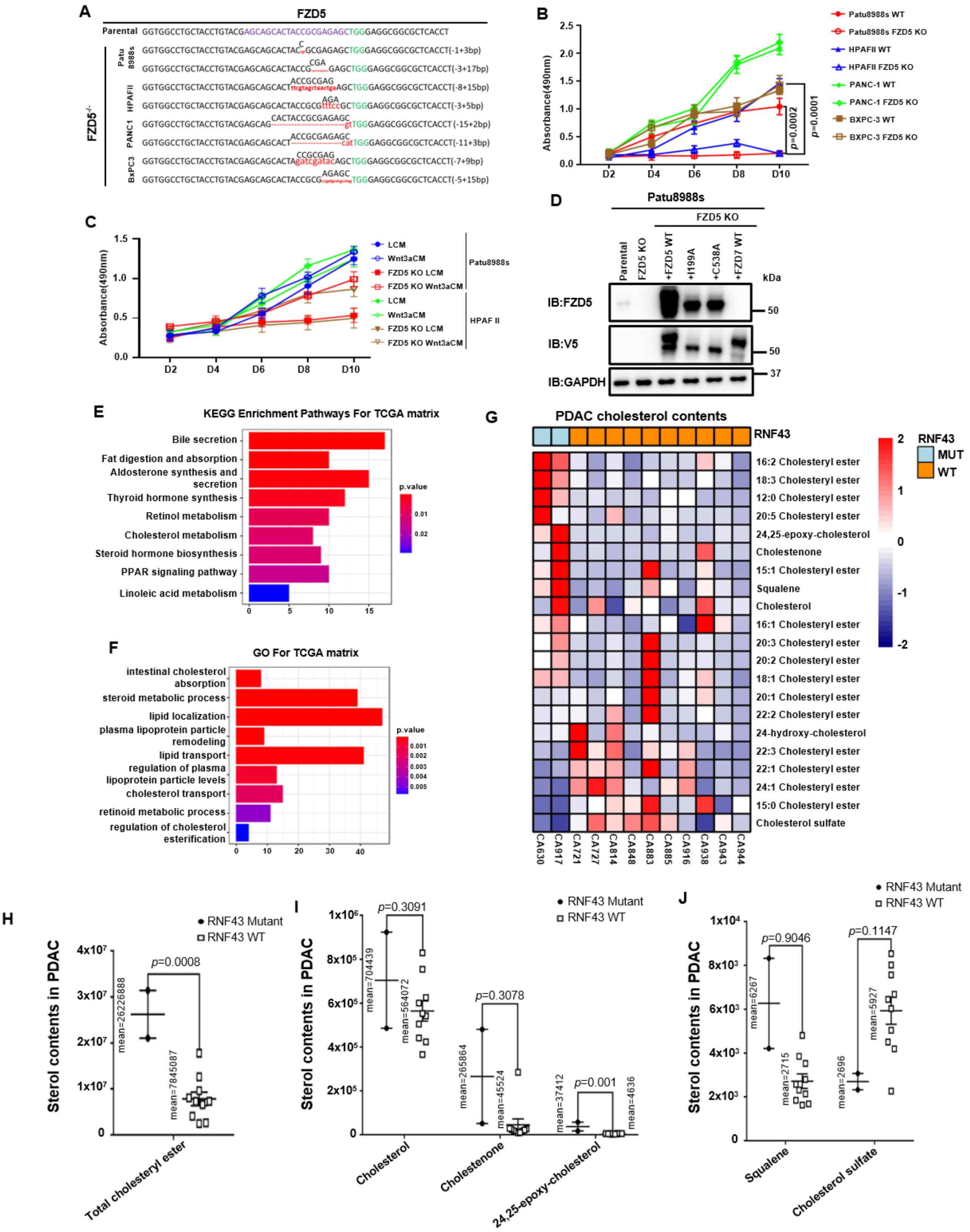
A
FZD5
Parental
FZD5-/-
Patu 8988s
HPAFII
PANC1
BxPC3
B
Absorbance(490nm)
Patu8988s WT
Patu8988s FZD5 KO
HPAFII WT
HPAFII FZD5 KO
PANC-1 WT
PANC-1 FZD5 KO
BXPC-3 WT
BXPC-3 FZD5 KO
p=0.0002
p=0.0001
C
Absorbance(490nm)
LCM
Wnt3aCM
FZD5 KO LCM
FZD5 KO Wnt3aCM
Patu8988s
HPAF II
D
Patu8988s
FZD5 KO
Parental
FZD5 KO
+FZD5 WT
+I199A
+C538A
+FZD7 WT
kDa
IB:FZD5
IB:V5
IB:GAPDH
E
KEGG Enrichment Pathways For TCGA matrix
Bile secretion
Fat digestion and absorption
Aldosterone synthesis and secretion
Thyroid hormone synthesis
Retinol metabolism
Cholesterol metabolism
Steroid hormone biosynthesis
PPAR signaling pathway
Linoleic acid metabolism
p.value
F
GO For TCGA matrix
intestinal cholesterol absorption
steroid metabolic process
lipid localization
plasma lipoprotein particle remodeling
lipid transport
regulation of plasma lipoprotein particle levels
cholesterol transport
retinoid metabolic process
regulation of cholesterol esterification
p.value
G
PDAC cholesterol contents
RNF43
MUT
WT
16:2 Cholesteryl ester
18:3 Cholesteryl ester
12:0 Cholesteryl ester
20:5 Cholesteryl ester
24,25-epoxy-cholesterol
Cholestenone
15:1 Cholesteryl ester
Squalene
Cholesterol
16:1 Cholesteryl ester
20:3 Cholesteryl ester
20:2 Cholesteryl ester
18:1 Cholesteryl ester
20:1 Cholesteryl ester
22:2 Cholesteryl ester
24-hydroxy-cholesterol
22:3 Cholesteryl ester
22:1 Cholesteryl ester
24:1 Cholesteryl ester
15:0 Cholesteryl ester
Cholesterol sulfate
CA630
CA917
CA721
CA727
CA814
CA848
CA883
CA885
CA916
CA938
CA943
CA944
H
Sterol contents in PDAC
RNF43 Mutant
RNF43 WT
p=0.0008
mean=26226888
mean=7845087
Total cholesteryl ester
I
Sterol contents in PDAC
RNF43 Mutant
RNF43 WT
p=0.3091
mean=704439
mean=564072
p=0.3078
mean=265864
mean=45524
p=0.001
mean=37412
mean=4636
Cholesterol
Cholestenone
24,25-epoxy-cholesterol
J
Sterol contents in PDAC
RNF43 Mutant
RNF43 WT
p=0.9046
mean=6267
mean=2715
p=0.1147
mean=2696
mean=5927
Squalene
Cholesterol sulfate

Supplemental Figure 5. Supporting information and assays showing RNF43-mutant PDAC has Fzd5 dependence and aberrant cholesterol metabolic pattern. A) Sanger sequence of Fzd5 KO cell lines. B) MTT cell growth assay for parental PDAC and their corresponding Fzd5 KO stable cell lines. Error bars mean ± SD, n = 3 replicates, by two-way ANOVA analysis. C) MTT cell growth assays of PDAC cell lines under LCM or Wnt3a CM treatment. n = 3 replicates. D) WB validation of the protein expressions in Figure 5A. E, F) KEGG E) and GO F) enrichments of the differentially expressed genes between RNF43-mutant and RNF43-WT PDAC in TCGA database. G) Lipid content heatmap of cholesterol metabolites showing that RNF43-mutant PDAC has a distinct cholesterol metabolic pattern compared to RNF43-WT PDAC. Each row represents a specific metabolite and each column represents a specific patient. H) Total cholesteryl ester content in RNF43-mutant PDAC is significantly higher than that in RNF43-WT PDAC. Error bars mean ± SEM, by two-tailed unpaired student's t-test analysis. I, J) Comparison of unesterified cholesterol metabolite contents between RNF43-mutant and -WT groups by histograms. Error bars mean ± SEM, by two-tailed unpaired student's t-test analysis.

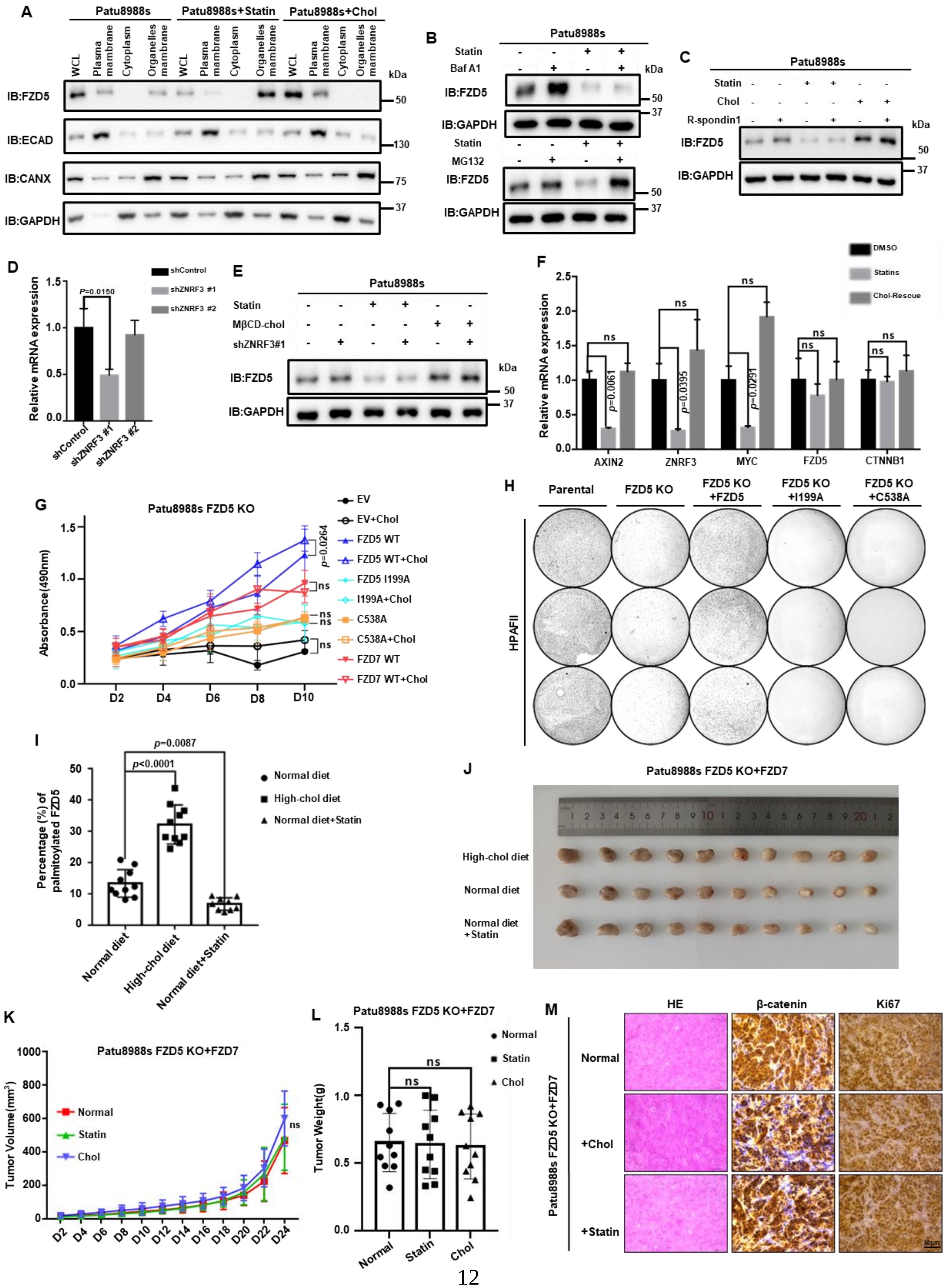

A
Patu8988s
Patu8988s+Statin
Patu8988s+Chol
WCL
Plasma mambrane
Cytoplasm
Organelles mambrane
kDa
IB:FZD5
IB:ECAD
IB:CANX
IB:GAPDH
50
130
75
37
B
Patu8988s
Statin
Baf A1
MG132
IB:FZD5
IB:GAPDH
C
Patu8988s
Statin
Chol
R-spondin1
IB:FZD5
IB:GAPDH
D
Relative mRNA expression
P=0.0150
shControl
shZNRF3 #1
shZNRF3 #2
E
Patu8988s
Statin
MβCD-chol
shZNRF3#1
IB:FZD5
IB:GAPDH
F
Relative mRNA expression
DMSO
Statins
Chol-Rescue
p=0.0061
p=0.0395
p=0.0291
ns
AXIN2
ZNRF3
MYC
FZD5
CTNNB1
G
Patu8988s FZD5 KO
Absorbance(490nm)
p=0.0264
ns
D2
D4
D6
D8
D10
EV
EV+Chol
FZD5 WT
FZD5 WT+Chol
FZD5 I199A
I199A+Chol
C538A
C538A+Chol
FZD7 WT
FZD7 WT+Chol
H
Parental
FZD5 KO
FZD5 KO +FZD5
FZD5 KO +I199A
FZD5 KO +C538A
HPAFII
I
p=0.0087
p<0.0001
Percentage (%) of palmitoylated FZD5
Normal diet
High-chol diet
Normal diet+Statin
J
Patu8988s FZD5 KO+FZD7
High-chol diet
Normal diet
Normal diet +Statin
K
Patu8988s FZD5 KO+FZD7
Tumor Volume (mm³)
Normal
Statin
Chol
ns
D2
D4
D6
D8
D10
D12
D14
D16
D18
D20
D22
D24
L
Patu8988s FZD5 KO+FZD7
Tumor Weight(g)
ns
Normal
Statin
Chol
M
HE
β-catenin
Ki67
Patu8988s FZD5 KO+FZD7
Normal
+Chol
+Statin
50μm

Supplemental Figure 6. Supporting assays showing cholesterol regulating RNF43-mutant PDAC growth via Fzd5-mediated Wnt/β-catenin signaling. A) Cellular fractionation assay of endogenous Fzd5 under different treatments in Patu8988s cells. B) Endogenous Fzd5 level test under Baf A1 and MG132 treatment in Patu8988s cells. C) Endogenous Fzd5 level test upon Rspo1 treatment in Patu8988s cells. D) Knockdown of ZNRF3 validation by RT-qPCR. Error bars mean ± SD, n = 3 replicates, by one-way ANOVA analysis. E) Endogenous Fzd5 level test upon ZNRF3 knockdown in Patu8988s cells. F) RT-qPCR assays of Wnt target genes, FZD5 and CTNNB1 under cholesterol starvation or rescue conditions in Patu8988s cells. Error bars mean ± SD, n = 3 replicates, by one-way ANOVA analysis. G) MTT cell growth assays of different Fzds rescued Patu8988s Fzd5 KO cell under normal or additional cholesterol conditions. Error bars mean ± SD, n = 3 replicates, by two-way ANOVA analysis. H) Colony formation assays of HPAF-II parental and Fzd5-KO cells with different rescue conditions. n = 3 replicates. I) Quantification of immunoblotting of S-palmitoylated endogenous Fzd5 percentage in the transplanted tumors shown in Figure 5K after APE-assay. Each data point represents an individual tumor. For high cholesterol diet and normal diet groups, n=10; for stain treated group, n=9. Error bars mean ± SD, by one-way ANOVA analysis. J) The PDAC tumors formed by subcutaneous implantations of Patu8988s Fzd5-KO Fzd7-rescue cells. From top to bottom, the rows represent high-cholesterol diet fed group, normal diet fed group and normal diet plus pravastatin fed group, respectively. n = 10 for each group. K) Subcutaneously implanted tumor size measurement by days. Error bars mean ± SD, n = 10 for each group, by two-way ANOVA analysis. L) Subcutaneously implanted tumor weight measurements after section at Day24. Error bars mean ± SD, n = 10 for each group, by one-way ANOVA analysis. M) Immunohistochemistry of subcutaneously implanted tumors and hematoxylin-eosin staining. ns: not significant.

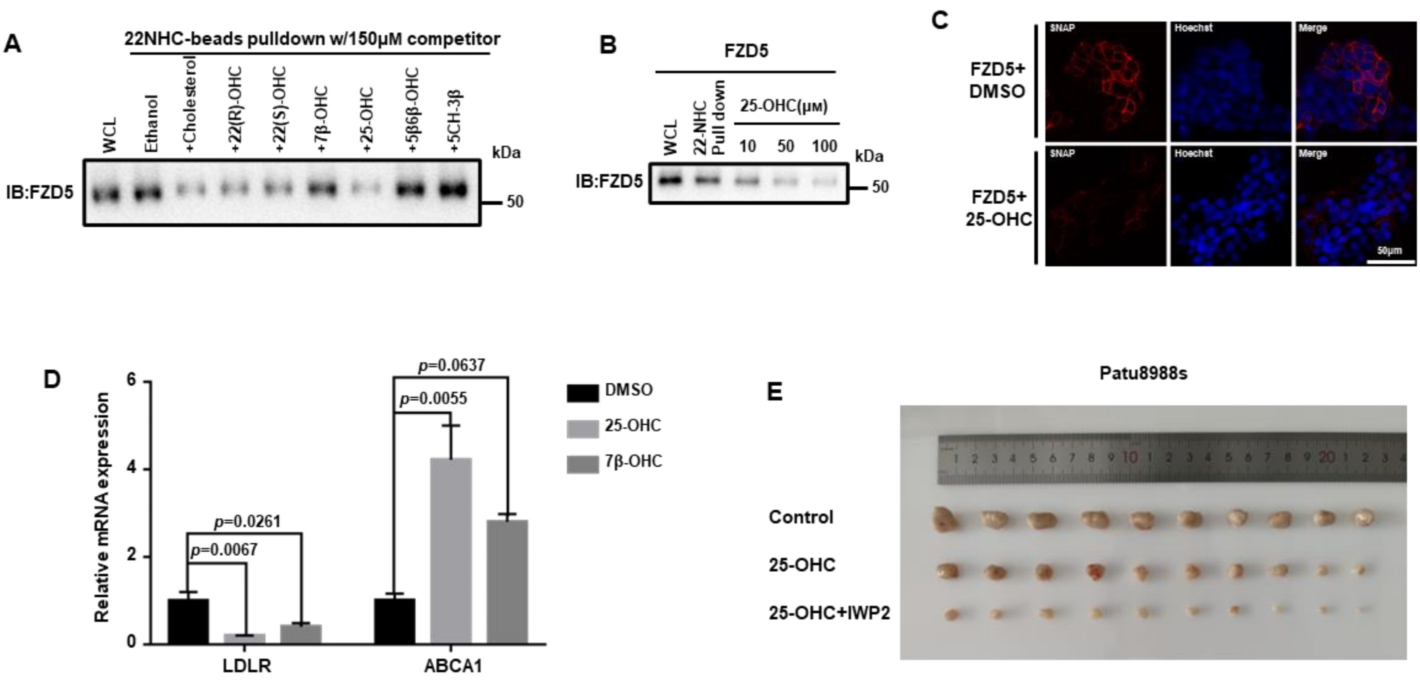
A
22NHC-beads pulldown w/150μM competitor
WCL
Ethanol
+Cholesterol
+22(R)-OHC
+22(S)-OHC
+7β-OHC
+25-OHC
+5β6β-OHC
+5CH-3β
kDa
IB:FZD5
50
B
FZD5
WCL
22-NHC Pull down
25-OHC(μм)
10
50
100
kDa
IB:FZD5
50
C
FZD5+ DMSO
FZD5+ 25-OHC
SNAP
Hoechst
Merge
50μm
D
Relative mRNA expression
p=0.0261
p=0.0067
p=0.0637
p=0.0055
DMSO
25-OHC
7β-OHC
LDLR
ABCA1
E
Patu8988s
Control
25-OHC
25-OHC+IWP2

Supplemental Figure 7. Supporting data showing 25-OHC treatment effects on Fzd5 PM localization and Fzd5-mediated PDAC growth. A) 22-NHC beads pulldown and competition assay. Endogenous Fzd5 is pulled down by 22-NHC and competed by various oxysterols such as 25-OHC. B) 22-NHC beads pulldown and competition assay of endogenous Fzd5 by different doses of 25-OHC. C) Confocal fluorescent imaging of SNAP-tagged Fzd5 on PM under normal and 25-OHC treatment conditions in HEK293T. All images are in the same scale. D) RT-qPCR assay of cholesterol metabolism genes under the treatment of 25-OHC and 7β-OHC in Patu8988s cells. Error bars mean ± SD, n = 3 replicates, by one-way ANOVA analysis. E) The PDAC tumors formed by subcutaneous implantations of Patu8988s cells. From top to bottom, the rows represent control group, 25-OHC treated group and 25-OHC+IWP2 treated group, respectively. n = 10 for each group.

Supplemental Table 1. PDAC patients' basic information

Supplemental Table 2. Mass spectrometry original data for the untargeted liposome analysis
